# Prospects of Understanding the Molecular Biology of Disease Resistance in Rice

**DOI:** 10.3390/ijms19041141

**Published:** 2018-04-10

**Authors:** Pankaj Kumar Singh, Akshay Nag, Preeti Arya, Ritu Kapoor, Akshay Singh, Rajdeep Jaswal, Tilak Raj Sharma

**Affiliations:** National Agri-Food Biotechnology Institute, Mohali 140 306, Punjab, India; pkmolbio@gmail.com (P.K.S.); nagaksh@gmail.com (A.N.); arya.preet9@gmail.com (P.A.); ritukapoor1985@gmail.com (R.K.); akshaybioinfo@gmail.com (A.S.); rajdeepjaswal52@gmail.com (R.J.)

**Keywords:** rice, disease resistance, breeding, biotic stress, marker assisted selection, signaling pathways, transcription factor

## Abstract

Rice is one of the important crops grown worldwide and is considered as an important crop for global food security. Rice is being affected by various fungal, bacterial and viral diseases resulting in huge yield losses every year. Deployment of resistance genes in various crops is one of the important methods of disease management. However, identification, cloning and characterization of disease resistance genes is a very tedious effort. To increase the life span of resistant cultivars, it is important to understand the molecular basis of plant host–pathogen interaction. With the advancement in rice genetics and genomics, several rice varieties resistant to fungal, bacterial and viral pathogens have been developed. However, resistance response of these varieties break down very frequently because of the emergence of more virulent races of the pathogen in nature. To increase the durability of resistance genes under field conditions, understanding the mechanismof resistance response and its molecular basis should be well understood. Some emerging concepts like interspecies transfer of pattern recognition receptors (PRRs) and transgenerational plant immunitycan be employed to develop sustainable broad spectrum resistant varieties of rice.

## 1. Introduction

Rice is one of the major cereal crops that fulfils more than 23% calorie needs of people worldwide and is a staple food crop for half of the world population living in Asia, where its cultivation covers approximately 92% of total acreage [1]. It is expected that the world population will exceed eightbillion by the year 2025 and to meet the increased global food and calorie demands, the total grain production has to be increased by up to 50% [2]. To fulfill this goal, it is imperative to decrease crop losses due to biotic and abiotic stresses [3]. Rice cropsare affected by around 70 pathogens, especially viruses, bacteria, fungi and nematodes that damage the crop severely and ultimately reduce yield [4]. Minimizing the losses caused by these diseases will thus increase total rice production. Deployment of resistance genes in rice is the best practice to manage diseases and reduce environmental damageby reducing the application of agro-chemicals. Development of disease resistance rice varieties has beenachieved by the implementation of plant breeding approaches including conventional breeding like introduction of exotic lines, backcross breeding, and modern biotechnological approaches such as molecularmarker assisted backcross breeding, gene pyramiding, etc.Molecular mapping of disease resistance genes, their cloning and generation of transgenic lines using genetic engineering methods are very promising approaches. These techniques explore the natural diversity present within the rice gene pools. However, natural diversity is not enough to continuously generate new resistance cultivars. To generate more variation, artificial mutations can be randomly created in the rice genome or the genes involved in the resistance mechanism can be directly targeted. Development of new resistant varieties is needed to protect crops from pathogens. Understanding themolecular basis of resistance against multiple pathogens is also important for the sustainable use of resistance sources under the pressure of global climate change.

We now understand that there are two tiers of defense in pathosystems: PAMP (Pathogen associated molecular pattern)-triggered immunity (PTI) and effector-triggered immunity (ETI) which act in a typical host plant against the pathogen attack. The PTI is the first level of defense barrier in the plant and this process begins by getting the perception or through sensing of microbe- or pathogen-associated molecular patterns (MAMPs or PAMPs). The second level of defense in the plant is the ETI which recognizes effector molecules delivered by pathogens inside host cell that have surpassed the PTI [5]. A primary characteristic of the plant innate immunity is activation of the PTI. The perception of PAMPs and MAMPs by pattern recognition receptors (PRRs) directs to the foundation of several downstream defense related signaling consequences. Thus, the virulence capability of a pathogen depends upon the deactivation of PTIs by its effector molecules [6]. The stimulation of PTI generates signaling networks of mitogen-activated protein (MAP) kinase, transcriptional recoding facilitated by transcription factors such as WRKY and formation of different reactive oxygen species (ROS) inside the host plant [7]. The ETI is activated by the binding of major *R* gene receptors of host plant and effectors of pathogen that activates host defense mechanism and generally leads to localized cell death surrounding the pathogen invasion [8]. In this review, molecular aspects of rice disease resistance, including the general approaches used for developing resistant plants, the genetic basis of host resistance, signal transduction pathways, defense mechanism, regulation of defense mechanism by regulatory elements and their perspectives in rice resistance will be discussed in response to fungal, bacterial and viral pathogens.

## 2. Mechanism of Plant Defense against Biotic Stresses

The defense system of plants comprises of multiple barriers ranging from outer barrier, like waxy cuticles to internal barriers like resistance and defense response genes. Plant resistance is entirely dependent on a network of signaling pathways involving innate immunity and a class of resistance genes [9,10,11]. Primarily, defense systems of plants can be categorized into two classes, basal defense and specific defense systems. The basal defense system checks the entry of pathogens in the plants andprovides immunity at the beginning of infection. This defense system is much effective against necrotrophic pathogens. The latter specific defense mechanism operates effectively against biotrophs and hemibiotrophs through hypersensitive response (HR) developed by programmed cell death at the surroundings of infection sites and thereby limits pathogen growth and disease development. This mechanism comes forward to control the pathogen when first level defense is breached [9,12]. The basal defense system or innate immunity is a generalized barrier which does not discriminate between pathogens, unlike the specific defense system which is mediated by a highly specific set of genes called resistance (*R*) genes. In the beginning of infection, waxy cuticle, and the cell wall restrict pathogens entry, however, most pathogens infecting plants like fungi harbor secretory proteins which degrade these barriers [13,14,15]. After the entry of pathogens inside the host cell, molecules get released (called Microbial/Pathogen Associated Molecular Pattern; MAMP or PAMP) which are composed of peptidoglycan, ergosterol lipopolysaccharide, and bacterial flagelin proteins. The innate immune system recognizes these proteins with the help of receptors present on the plasma membrane of host cell called pattern recognition receptors (PRRs) to further restrict the progress of infection providing MAMP-triggered immunity (MTI). PRRs also detect molecules that are native components of the host which, however, get released when damage is done by pathogens and are known as damage-associated molecular patterns (DAMP). The binding of these components also activates PTI and downstream defense responses [16,17]. Overall, the recognition of MAMP/PAMP or DAMP leads to the activation of PTI causing different reactive oxygen species (ROS) production, initiation of mitogen-activated protein (MAP) kinase activity and various transcription factoractivation restricting the spread of pathogens completely [7].

Several studies have proven that expression of defense response (DR) genes like *Chitinase*s and *Phenylalanineammonia-lyase* (*PAL*) can directly correlate with host resistance [18,19]. Rice germin-like proteins (OsGLP) are a class of DR genes, present in a QTL along with *R* genes and are potentially associated with resistance of rice, as silencing of these genes increased the susceptibility against two major fungal pathogens, sheath blight and rice blast [20]. *OsPAL4* is associated with broad spectrum disease resistance in rice [19]. A LysM receptor like kinase (RLK), *OsCERK1,* regulates cytoplasmic *OsRLCK176* and *OsRLCK18*5, recognizes chitin and peptidoglycans activating immune signaling pathways in rice against *Magnaporthe oryzae* and *Xanthomonas oryzae* pv. *oryzae* (*Xoo*). *OsCERK1*, *OsRLCK176* and *OsRLCK185* follow the same signaling pathways; however, *OsRLCK176* and *OsRLCK185* function downstream in the signaling pathway governed by *OsCERK1*. The phosphorylation of *OsRLCK185* is mediated by *OsCERK1* to activate MAPK pathways. The *Xoo* effector Xoo1488 inhibits the phosphorylation of *OsRLCK185* causing pathogen resistance [21,22]. Three *RLKs* and *OsWAK* genes in rice are transcriptionally co-regulated as these *RLKs* are known to be required for enhanced resistance against *M. oryzae* [23]. Transcriptome and micro RNA analysis of two varieties of rice (resistance versus susceptible) revealed different signaling pathways as well as variable micro RNA expression causing change in innate immunity against *M. oryzae* [24]. A lectin *RLK* from rice is also found to show association with innate immunity and interacts with depolymerizing factor of actins [25].

Most of the above-mentioned examples are mediated by pattern recognition receptors (PRRs). Basically, PRRs belong to receptor like kinase (RLK) and receptor like protein (RLP) classes. The RLK has a single membrane-embedded domain, a kinase domain present intracellularly and a domain present extracellularly for sensing ligand molecules, whereas RLP has a membrane-embedded extracellular region but lacks kinases region [26]. RLPs are structurally and functionally similar to Toll like receptors (TLR) of animals [27]. The extracellular ligand-sensing domain of PRR is generally rich in leucine repeats [28,29]. In contrast to animals, plants are sessile and are affected by biotic, abiotic and multiple other factors, therefore, plants may have higher numbers of RLK and RLP than animals. Approximately, 640 RLKs and 90 RLPs are reported in rice [30].

PAMP marker detection which lead to plant immunity is a widespread and effective mechanism to restrict most of the pathogens. However, in spite of this, multiple pathogens including fungi and bacteria possess a varietyof proteins that enter the plant and manipulate innate immunity of hosts whileremaining undetected. In this scenario, a second line of defense comes into action that includes resistance (*R*) gene, composed of mainly nucleotide binding site (NBS), Leucine Rich Repeat (LRR) domains and other domains [5]. The *R* genes impart resistance against pathogens by recognizing secreted effector proteins called avirulence (*Avr*) genes and provide immunity, i.e., effectortriggered immunity (ETI). This resistance provided by *R* genes could be the result of either direct interaction with the *Avr* gene or indirect interaction that involves other mediator proteins. The indirect interaction of *R*-*Avr* involves finding modifications of other host proteins (effector targets) that interact with the effectors. By this interaction, a cascade of defense response networks are activated leading to HR, restricting pathogen growth at the infection site. The immunity provided by the innate immune response and *R* genes is altogether mediated by a complex network of signaling pathways leading to the activation of defense-responsive genes such as pathogenesis-related genes (*PR*), reactive oxygen species (ROS), *glucanases*, *chitinases*, secondary metabolites, physiology of stomata closure, and deposition of callose and lignin. The products of these genes largely act at the protein levels against fungal and bacterial pathogens and are involved in regulation at the mRNA level. In contrast to the defense mechanism against viral diseases, they generally actat the RNA level [31,32,33,34].

## 3. Resistance Gene Architecture and Resistance Hypothesis

Plant resistance *R* genes are generally a multi domain gene family having NB-ARC (nucleotide-binding adaptor shared by APAF-1, R proteins, and CED-4) as a primary domain and Leucine Rich Repeats (LRR) as an associated domain. The nucleotide binding site (NBS) domains play a key role in host defense mechanisms [35,36,37]. The LRR domain has a role in the recognition of pathogen gene products [9,38]. The NBS-LRR domains are functionally dependent on each other and are under simultaneous pressure [39]. In NBS domains, multiple motif sequences like MHDV, RNBS-A, RNBS-B and GLPL have also been reported, that are crucial in maintaining the function of the NBS domain [40]. In eukaryotes, these are collectively called NLRs (Nucleotide binding domain, Leucine-rich Repeat). In rice, approximately 438 putative *NLR* genes have been reported [41,42].

Various models have been given to describe the interaction of *R*- and *Avr*-genes. The very first model put forward was the gene-for-gene hypothesis. According tothis hypothesis, the *R* gene product of the host plant interacts directly with its corresponding *Avr* gene product of the pathogen [43]. This hypothesis was followed by multiple cases of *R*-*Avr* interactions, i.e., a single *NLR* gene recognizes its counterpart *Avr* effector and imparts resistance to the pathogen [5,44]. This type of direct interaction has been reported in the case of the rice blast resistance protein Pi54 and its counterpart Avirulence protein, AvrPi54 [45]. Indirect interaction between *R*- and *Avr*-gene products is called the Guard–Guardee model, in which *R*-genes monitor the target and interaction of effector molecules and modification of this target acts as an indicator of pathogen attack. Two proteins of *Solanum lycopersicum* (tomato), Pto and Prf follow this mechanism to interact with the AvrPto proteins of the bacterial pathogen *Pseudomonas syringae* [46], however, recognition of effectors in multiple pathogens by indirect means lead to a modified hypothesis called the decoy model which states that an effector protein may have generated multiple targets in the host (decoy proteins) by duplication events [47]. The decoy model has been reported in multiple studies for effector *Pto*, *Bs3*, *RCR3*, and *RIN4* [47]. Recently, in some studies, the multiple decoy domain has also been found to show a link with NLR proteins [48,49,50]. The NLR proteins with decoy domain were firstly reported in poplar tree plants having BED domain as a decoy domain [51]. This type of *R* gene function was reported in a study in which sugar transferase, BED-type zinc finger andcarbohydrate esterase 4 superfamily acted as decoy domains in nine putative *R* genes and in a resistance gene (*Xa1)* of rice, and further speculated that these domains may integrate in *R* genes to functions as sensors [52].

This type of *R* gene action has also been validated in rice-*M. oryzae* pathosystem [48,53,54], where RGA5 and Pik-1, two proteins of rice contain RATX1/HMA domain whichare similar to gene *Pi21*, interact physically with two effectors, *Avr*-*Pia* as well as with *Avr*-*Pik* of *M. oryzae*. In another study, Kroj et al. [55] reported that the NLR and decoy domain association is extensive and common in plants. Actually, they mined 31 plant genomes for finding NLR with putative decoy domains and further evaluated the role of NLR-BED decoy domain protein in rice for resistance against *M. oryzae* using overexpression and knockout mutant analysis. Although few R proteins arealready known to contain decoy domains, e.g., the NLR CHS3 has zinc-finger domain [56], the rice resistance protein Pi-ta contains a thioredoxin domain [57], Xa1 comprises a BED domain [58] and Pi54 contains a unique zinc finger (NFX) domain [42,59]. Sarris et al. [60] also found that the fused domain with multiple NLR proteins may act as bait for pathogen effectors as these domains areknown to interact with pathogens.

## 4. Signal Molecules and Their Networks Involved in Rice Defense Response

The plant cell initiates a network of defense signaling cascades on the perception of pathogen elicitors through PRRs and disease resistance (R) proteins.This results in a defense response to confine the pathogen within the infection site. The defense response includes changes in membrane permeability and ion fluxes (Ca^2+^, K^+^, H^+^), generation of ROS and NO, production of pathogenesis responsive (PR) proteins (glucanases, chitinases, defensins), cell wall strengthening (callose and lignin deposition), phytoalexin synthesis and activation of kinase cascades accompanied by hypersensitive response (HR) [5]. This defense response is mediated by cross-communicating sets of endogenous signal molecules, including phytohormones, ROS and NO which in turn are regulated by numerous transcription factors [61]. These Jasmonic Acid (JA), Salicylic Acid (SA), and Ethylene (ET) are the archetypical players in the regulation of defense signal transduction cascades.

### 4.1. Mitogen-Associated Protein (MAP) Kinase and Ca^2+^ Signaling

Following pathogen perception, plant receptors activate ion channels, GTP binding proteins, and kinases, which in turn activate secondary messengers to amplify signals to the diverse set of downstream cascades. The MAP kinase cascade is the prevalent component of plant defense signaling, mainly in the PTI signaling pathway. It comprises of three interlinking proteins where MAPKKK stimulates MAPKK which in turn stimulate MAPK. Thus, activated MAPK stimulates TF and other downstream signaling molecules through trans-phosphorylation reactions [62]. A total of 17 MAPKs have been identified from *O. sativa*; however, only five MAPKs (*OsWJUMK1*, *OsMAPK4*, *OsMAPK5*, *OsMAPK6*, and *MAPK12 OsBWMK1*, for blast and wound induced MAP kinase) have been characterized for their function [63]. Ca^2+^ influx is another essential and conserved event in the plant defense responses which stimulates intracellular signaling directly or through Ca^2+^ sensors. The Ca^2+^ sensors can be either Ca^2+^-dependent protein kinases (CDPKs), chimeric Ca^2+^/calmodulin-dependent protein kinases (CCaMKs) or CDPK-related kinases (CRKs) [64]. Activation of typical CDPKs stimulates the CCaMKs, enzymes or TFs in the downstream processes of defense response. The CDPKs and MAPKs act either synergistically or independently in the innate immune responses.

### 4.2. Role of ROS and NO in Rice Defense Signaling

The generation of reactive oxygen species (superoxide, O^2−^ and H_2_O_2_ accompanied by oxidative burst) is a conserved early response in plant defense signaling cascades, generally accompanied by programmed cell death/hypersensitive response during plant–pathogen interaction. The NADPH oxidase, peroxidase and oxidases are the sources of ROS generation in cell organelles, including mitochondria, chloroplasts and peroxisomes [65]. This oxidative burst drives the cell wall reinforcements or cell wall strengthening for cellular protection. The pathogen avirulence factors induce the production of nitric oxide (NO) which potentiate the induction of defense-related gene expression and secondary metabolite synthesis [66], thereby, playing a key role in disease resistance in plants.

### 4.3. Phytohormone-Mediated Signaling

**Figure 1:**
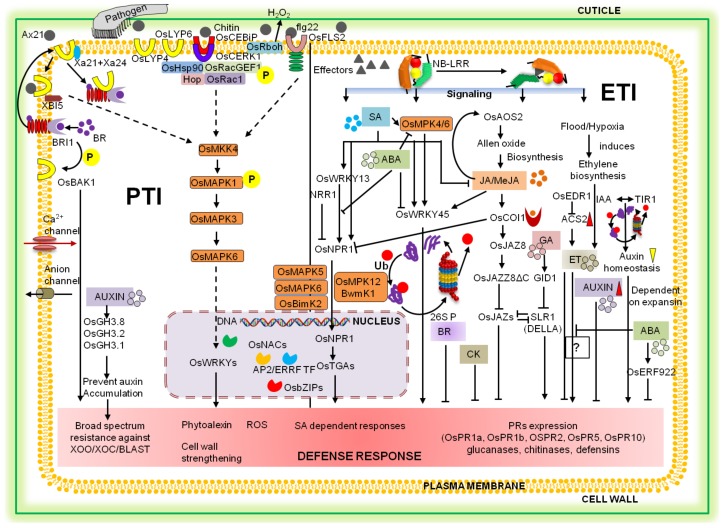
Schematic representation of rice defense signaling cascades. Following the pathogen perception bypattern recognition receptors(PRRs) and R proteins, the rice plant initiates the diverse set of signaling cascades at different levels (PAMP-triggered immunity (PTI), left side and ETI, right side) involving numerous signal molecules, viz. ROS, NO, MAPKs, CDPKs, phytohormones to trade-off the pathogen invasion. In case of PTI, the host cell recognizes the common molecular pattern associated with most of the pathogens using PRRs (OsLYP6, OsFLS2, OsCEBiP, OsCERK1, Xa21, Xa24) and initiates MAPK kinase cascades (OsMKK4–OsMAPK6) that actually activate host defense responses via various transcriptional regulatory factors (OsWRKYs, OsNACs, OsNPR1, OsTGAs, OsbZIPs). However, PTI is suppressed by pathogen effectors, where they are encountered by the resistance genes (*NBS*-*LRRs*) that lead signaling to activate defense responses through phytohormonal activities. The archetypical defense pathways, SA and JA/ET pathways, mainly antagonistic to each other, are responsible for resistance against biotrophs and necrotrophs, respectively. The defense response includes production of PR proteins (glucanases, chitinases, defensins), production of ROS and NO, change in ion fluxes (Ca^2+^), cell wall strengthening (callose and lignin deposition) to confine the pathogen dissemination and disease development. GA, CK and Auxin act as negative regulators of plant innate immunity. BR prompts or suppresses disease susceptibility based on pathogen lifestyle or colonization. Furthermore, abscisic acid (ABA), well-known in abiotic stress tolerance, plays an ambiguous role, i.e., is both a positive and negative regulator of rice disease resistance based on the type and stage of infection; however, it predominantly actsas a negative regulator. The abbreviations used in the figure above represent viz. SA-salicylic acid; JA-Jasmonic acid, MeJA-Methyl Jasmonate; GB-Gibberellins, BR-Brassinosteroid; ET-Ethylene, CK-Cytokinin; ABA-Abscisic Acid, OsNPR1-Non-expressor of PR1 (NH1, NPR1 homolog1); OsCOI1-Coronatine Insensitive1 (JA receptor); OsJAZ8-Jasmonate ZIM domain protein, HPL3-Hydroperoxide lyase; ACS2-Enzyme for ET biosynthesis (ACC Synthase); OsEDR1-Enhanced Disease resistance 1 (TR1-like kinase); SLR1-slender rice1 (DELLA protein); GID1-encodes GA receptor; BRI1-BR Insensitive 1 (RLK) BR receptor.

Plant immunity follows a binary model for phytohormone signaling, including SA- and JA/ET-dependent pathways, which interact in a mutually antagonistic way to trade-off pathogens, which is similarin rice. Moreover, SA often induces resistance against hemibiotrophs and biotrophs whereas necrotrophs are usually deterred by JA/ET-dependent cascades [67]. However, rice crop reveals new insights and unique features ofplant immunity as compared to *Arabidopsis* [68]. The endogenous level of SA often increases substantially for induction of the pathogenesis-related (PR) proteins and defense response. However, involvement of the SA in rice defense mobilization was more reliantupon SA signaling rather than the variation in its endogenous level or its *de novo* synthesis [68,69]. The SA pathway in rice shares *OsNPR1/OsNH1*, *OsTGAs*, *OsWRKY13* [70] and *OsWRKY45* (autoregulated) [71] as downstream signaling components which in turn induce PR protein accumulation (Figure 1) to confer resistance against rice blast and bacterial blight. JA is often considered to be predominantly effective against necrotrophs but enhances susceptibility to biotrophs. However, monocots including rice did not completely follow this dichotomy [72,73]. Accumulating evidence showsthat JA is a powerful signal to trade-off hemibiotrophs and biotrophs including *X. oryzae* and *M. oryzae* in rice [74,75] as well as to defend-off necrotroph including *R. solani* [75]. Moreover, ET functions synergistically with JA signaling and acts as a two-faced regulator in rice defense signaling since its application enhances disease resistance against pathogens such as rice blast, whereas it leads to disease susceptibility in case ofbacterial blight depending on pathogen’s infection biology [76].

There is extensive crosstalk between various phytohormones in rice against biotic stresses. For example, SLR1 (a rice DELLA protein) is an important regulator of GA signaling and suppresses GA biosynthesis. In addition, SLR1 mediates resistance against hemibiotrophs and biotrophs, but not necrotrophs in rice through integration and amplification of SA- as well as JA/ET-dependent signaling [77]. Moreover, cytokinin (CK) acts synergistically with SA signaling and enhances resistance against hemibiotrophic and biotrophic pathogens [78]. Moreover, auxin accumulation enhances disease susceptibility in rice. For instance, over-expression of *OsGH3.8*, *OsGH3.1*, and *OsGH3.2* prevents auxin accumulation and is responsible for enhanced broad spectrum resistance against *Xoo*/*M. oryzae*/*Xoc* pathogens in innate immunity [79]. Auxin signaling stimulates *OsWRKY31*-depending expression of defense-related and auxin responsive genes which in turn suppress the auxin sensitivity and enhances the resistance against rice blast [80]. Furthermore, brassinosteroids (BR) are reported to be involved in modulation of innate immunity based on BAK1-dependent and BAK1-independent defense response [81]. Surprisingly, abscisic acid (ABA), often known to be involved in abiotic stress tolerance is a negative regulator of biotic stress response. For example, exogenous applications of ABA and ABA biosynthesis inhibitor suppress resistance and reduce susceptibility against cold stress and rice blast, respectively [82,83]. In summary, the defense signaling network is a complex and cross-communicating network of interaction between signal molecules, including ROS, NO, MAPK, CDPKs and phytohormones, and any impairment in this signaling network enhances the chance of disease susceptibility in rice plants.

## 5. Role of Regulatory Elements in Rice Disease Resistance

Plant responses towards biotic stresses are specially facilitated by the action of different phytohormones in a network of signaling pathways, in which salicylic acid (SA) reaction to stress are an antagonistic way of the responses generated by jasmonic acid (JA)/ethylene transport (ET) pathways. In these signaling networks, the SA pathway is mainly associated with the responses developed against biotrophic pathogens. However, the responses induced by necrotrophic pathogens are synergistically regulated by JA and ET pathways. This synergistic action is also involved in plant defenses against various insect pests [84]. Some chemicals are also known to induce SA-derived responses in the plant (like probenazole, benzothiadiazole (BTH) which increase the level of SA in rice plants to mimic as biotrophic pathogens) [85]. The SA pathway plays a key role in the systemic acquired resistance (SAR) defense mechanism by activation of *NPR1* gene. Afterward, this gene provides broad spectrum resistance response against the pathogens. However, it does not provide immunity to the plant alone; several signaling transductions simultaneously work altogether to produce the resistance response [86]. *NPR1* protein is active only in its monomeric form, otherwise, it is present in a dimeric inactive form and the monomeric change is induced by the SA pathway, in which SAs breakdown the disulphide bonds of dimeric protein to generate monomers [87]. This protein directly interacts with transcription factors (TFs) of the *TGA* gene family to activate defense response [88]. Nevertheless, it also negatively regulates SAR in the plants with the help of *WRKY*-TFs [89]. For instance, *OsNPR1/NH1* functions as a repressor or negative regulator to provide resistance in rice against bacterial blight by manipulating expression levels of other defense- and photosynthesis-related genes [69,90,91]. The key *WRKY*-TFs involved in the the SA-derived resistance response is *WRKY45*, which is induced by SA activity and treatment of plants with BTH. However, it has been reported that *WRKY45* works independently and is not directly related to the *NH1* (homolog of *NPR1*) in rice [92]. This TF is reported to activate resistance response in transgenic lines against *M. oryzae* after treatment with BTH. Its physical interaction with a panicle blast resistance gene *Pb1* has been reported to provide broad spectrum resistance to *M. oryzae* [93]. In many studies, it has been concluded that *WRKY45* plays a major role in defense response of rice against rice blast and bacterial blight pathogens [92,94]. However, rice *WRKY45* has two different alleles (*OsWRKY45*-*1*, *OsWRKY45*-*2*) in indica and japonica subspecies and both the alleles were found to show resistance to *M. oryzae*, but, not to bacterial diseases [94]. The allele *OsWRKY45*-*1* does not provide resistance to the bacterial pathogens *Xoo* and *Xoc*, whereas, *OsWRKY45*-*2* shows direct involvement in bacterial resistance [94]. 

Very few TFs act as master regulators in responses to various biotic stresses in plants besides *WRKY45* [94]. *WRKY13* is another major regulatory factor to transmit signals from *WRKY45* to downstream functioning *WRKY*-TFs such as *WRKY42*. *WRKY13* follows the SA-pathway-dependent disease resistance mechanism and shows association with resistance responses against *M. oryzae* and *Xoo* [95,96]. Furthermore, this regulatory factor has two cis-elements (*PRE2* and *PRE4*), which are identified as pathogen-responsive regulatory elements [70,97]. Overall, *WRKY13* regulation is mediated by the SA pathway and pathway-related genes, while JA pathway-related signals are suppressed during this regulation [95]. Several *WRKY*-TFs other than master regulators have been identified and characterized; these play a role in rice resistance response for *M. oryzae* [79,98,99,100,101,102], *X. oryzae* [103] and *Rhizoctonia solani* [104,105]. Hence, the *WRKY-*TFs family is one of the major transcription regulatory elements involved in rice resistance response through SA, JA, ET and ABA signaling networks, because all these signals are interlinked and have crosstalk between them.

Several other major and minor non-*WRKY*-TFs have also been identified in rice. *NAC* is a major regulatory element that has been recognized to induce and impart resistance response in rice plant through the activation of *PR* genes. Many *NAC*-TFs are reported to provide innate immunity in rice, mainly against *M. oryzae.* Several findings have been documented with reference to *NAC*-TF induced resistance in rice and these are, *ONAC122*, *ONAC131*, *OsNAC6*, *OsNAC19*and *OsNAC111* [106,107,108,109]. Another transcription factor, basic leucine Zipper (*bZIP*), was found to regulate the signal transduction- and the defense-related genes in rice, thus restricting *M. oryzae* infection [110]. In a study based on *bZIP*, *OsBBI1*, a *bZIP* gene was identified that regulates the resistance spectrum in rice for diverse groups of *M. oryzae* by altering the first level of defense mechanism in host plant [111]. In addition, *OsBRR1* was also found to have resistance regulating action in rice against a certain set of *M. oryzae* isolates [112]. Therefore, various types of transcription factors play an important role in inducing immune response in rice via positive or negative gene regulation. C_2_H_2_-type TF was discovered to regulate rice resistance in a manner of non-race-specific to *M. oryzae*, while another *AP2/ERF* (*OsEREBP1*) controlled the rice resistance mechanism in a specific manner against *X. oryzae* [113,114]. Expression of ethylene responsive transcription factors *OsBIERF1*, *OsBIERF3* and *OsBIERF4* was induced by infection with *M. oryzae*, suggesting their role in biotic stresses [115]. Other rice TFs associated with resistance response towards biotic stresses are *OsDR10*, *OsGAP1*, *OsRac1*, for *X. oryzae* [116,117,118], *OsAOS2*, *Rir1b* for *M. oryzae* [119,120], *OsPLDβ1*, *OsDR8*, *OsSBP* for *X. oryzae* and *M. oryzae* [88,121,122]. Interestingly, some regulatory elements in rice like *Rir1b* are expressed at a much higher level when rice is infected with its non-host pathogen *Pseudomonas syringae* pv. *syringae* and that accumulated transcript of the *Rir1b* gene produced an enhanced resistance response in rice against *M.oryzae* [120]. Furthermore, *OsRac1* is reported to be essential for innate immunity in rice to *M. oryzae*, however, it also directly interacts with *Pit*, an NBS-LRR resistance gene. This interaction enhances effectiveness of the resistance in rice to *M. oryzae* [123]. Another *OsGH3.1* regulatory element is reported to help rice by providing resistance against a fungal infection [124].

## 6. Breeding Approaches to Control Diseases in Rice

The genus *Oryza* contains 23 species, of which only twoare cultivated and the rest 21 are wild types [125]. *Oryza sativa* (Asian rice) and *O. glaberrima* (African rice) are the cultivated species of rice and *O. sativa* is the only species of rice grown worldwide, whereas *O. glaberrima* is limited to some parts of West Africa. The *O. sativa* also has two subspecies indica and japonica. Japonica subspecies are further grouped into temperate and tropical rice with their growing suitability, and tropical japonica is known as javanica, which is considered as a separate subspecies of *O. sativa*. Now, it is a well-established fact that genetic diversity iskey to the improvement of any crop plant by transferring the genes for useful traits from the land races and wild-relatives into elite cultivars. Thus, natural genetic diversity is a major resource for various plant breeding programs, including resistance breeding for biotic stresses. Only 6 of 21 wild species, namely *O. glumaepatula*, *O. breviligulata*, *O. meridionalis*, *O. longistaminata*, *O*. rufipogon and *O. nivara*, and two cultivated *Oryza* species *O. sativa* and *O. glaberrima* have been utilized in rice crop improvement programs via breeding techniques, because they make the primary gene pool (AA genome) and share genetic constituents without any hindrance [126,127,128]. Besides, two additional gene pools, secondary and tertiary are reported for rice, but they are not suitable for traditional breeding programs, however, several useful traits taken from them have been exploited with the help of biotechnological tools and other approaches in conjunction with the breeding programs. Conventional breeding methods are the same for all kinds of agronomically important traits used in rice improvement programs. There are two types of resistance reported in the plant against biotic stresses, i.e., partial and complete resistance [129]. Partial resistance is governed by more than one gene and also called quantitative resistance or polygenic resistance. It provides non-race-specific resistance against the pathogens through quantitative resistance loci [130], while complete resistance is controlled by a single gene. It possesses a qualitative character and provides race-specific resistance against the pathogens. Nevertheless, sometimes, a single gene acts both ways and provides complete as well as partial resistance towards pathogens. Both cases of resistance have been reported in rice to control various plant pathogens. Major techniques employed in the rice resistance breeding are given below.

### 6.1. Introduction of Resistant Exotic Lines

Introduction of new germplasm lines and local landraces is an alternative way to augment the genetic diversity of crop plants in a confined area where local germplasm lacks resistance to biotic stresses [131]. It is a process to bring foreign genetic material for evaluation and utilize selected agronomically superior lines as a resistant variety in such areas where a certain pathogenic strain causes disease to all the local cultivars. It is a quick method to resolve the problem, but bringing genetic material across the globe is very tough due to country-wise laws that make this method very limiting in disease resistance breeding programs. However, the introduction of exotic lines was reported to be an efficient method for development of disease resistant cultivars [132]. For instance, Thippeswamy et al. [133] reported that the most of exotic rice germplasm taken from the International Rice Research Institute (IRRI) have shown resistance to local South Indian races of blast fungus, indicating high potential of this method in resistance breeding.

### 6.2. Introgression of Resistance Genes

Management of diseases in rice through the use ofresistant cultivars is one of thebest ways to tackle the problem because it reduces the pesticide application in rice fields, hence, minimizing the cost of crop production and subsequently lowering agrochemical pollution in the fields. The deployment of resistant cultivars to various rice diseases has already been published [134,135,136]. Land races and wild relatives of rice are usually used as sources for introgression of a new resistance gene into elite cultivar. Nevertheless, introgression of a resistance gene is challenging with conventional breeding methods because of the linkage-drag of undesirable traits that is very hard to break in spite of many generations of back-crosses [137]. Backcross breeding is normally employed for the introgression of resistance genes by inserting single disease resistant genes into a susceptible high yielding elite cultivar. Many near isogenic rice lines (NILs) have been generated through backcross breeding and employed in the development of resistant rice variety. Plant breeders also develop disease-resistant hybrids and cultivars using a conventional hybridization method. In this method, combining the genes of agronomically important traits, including disease resistance from different sources are practiced to improve the crop plants. Several cultivars of crop plants have been developed to enhance the disease resistance using this method [138].

### 6.3. Pyramiding of Resistance Genes

Gene pyramiding is one of the most effective plants breeding strategies for achieving multiple and durable resistance against many plant diseases. In this breeding method, genes pyramided from different genotypes in single cultivars offer long-term resistance towards pathogens and has turned into a plant breeders’ tool to generate broad-spectrum disease-resistant cultivars which restrict emergence of various pathogen races. Many different approaches such as composite breeding [139,140], synthetics crosses, and multiline crop breeding [141,142,143] have been utilized for developing gene pyramided lines of crop plants against various biotic stresses. Multiline breeding is also an important method of resistance improvement in crop plants [141,142,143]. It is also known as the “dirty crop” breeding method as in this approach, an elite cultivar is improved by developing many isogenic or near isogenic lines (NILs). The NILs are created by transferring a single resistance gene from various sources in different single plants by several rounds of backcrossing and, thus, each line has a separate resistance gene. Afterward, the NILs are bulked and the bulkedlines are called multiline as they have many lines containing separate resistance genes. This tool assists in gene pyramiding to pool several resistance genes together into a single genotype offering durable resistance. These lines are morphologically similarplants that may be genetically less different [141]. Rice breeders have generated many blast disease-resistant cultivars using this breeding method [143,144,145].

## 7. Molecular Breeding Approaches for Disease Resistance

There are various problems associated with the classical breeding methods for development of resistant cultivars; they require longer time periods, are more effort and labor intensive, they transfer undesirable genes along with the resistance genes by hybridization, they exhibit frequent resistance breakdown due to high mutagenic ability of pathogen and develop new pathogenic races. There are limited natural sources for resistance, and poor understanding ofthe resistance mechanism in the traditional breeding methods. Hence, there isa necessity to advance innovative and more efficient methods to overcome these problems. With the improvement of molecular genetics and biotechnological knowledge, several modern tools have been derived for this rationale. Considering these facts, some modern techniques of plant breeding related to disease resistance in rice against various fungi, bacteria and viruses have been employed.

### 7.1. Marker-Assisted Selection and Mapping of Resistance Genes

Traditional plant breeding for resistance is mostly dependent upon the phenotypic symptoms which are strongly related to environmental conditions in the field, consequently, newly emergingvirulent race of pathogens cannot be easily identified, and the introduction of improved resistant varieties cannot be reliable [146]. Unlike conventional breeding, marker-assisted selection (MAS) is very effective in disease resistance breeding because major resistance in the plant is operated by single or a few genes [130]. It is very useful in such host–pathogen interaction where both resistance (*R*) gene and avirulence (*Avr*) gene show interaction in the manner of gene-for-gene fashion [147]. Molecular markers increase the efficiency of conventional breeding by choosing the markers that are tightly linked to the desired traits. Similarly, molecular markers are also beneficial for identifying loci that operate quantitative traits [148]. Molecular markers, SSR, SNP, EST, RAPD, AFLP and RFLP are normally used to map several major resistance genes and QTLs in rice (Table 1 and Table 2).

**Figure 2:**
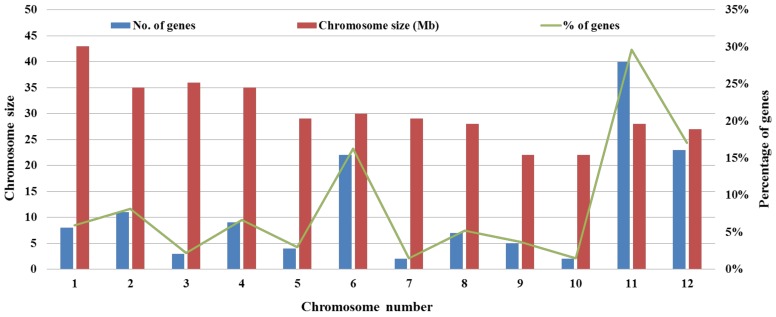
Chromosome-wise distribution of major resistance genes identified from rice. Numbers 1–12 represent the chromosome of rice. Percentage of resistance gene sharing on each chromosome is shown in green solid line, while blue and red bars represent number of resistance genes on each chromosome and chromosome size (Mb), respectively.

In this regard, resistance to blast disease has been recorded in rice as qualitative and quantitative [4]. Over 100 genes giving complete/partial blast resistance in rice have been documented [1,306]. Moreover, most of the blast *R* genes were characterized by a map-based cloning approach. For instance, *Pi54* is a major blast *R* gene and provides broad-spectrum resistance against many races of *M. oryzae*; it was cloned by map-based cloning approach from chromosome 11 of rice [238]. Similarly, Kumar et al. [257] also identified a broad-spectrum blast resistance gene *Pi42(t)* that was mapped on the chromosome 12 using the molecular marker techniques, but it is yet to be cloned and characterized. Both chromosomes 11 and 12 are the major blast resistance-gene-containing chromosomes. Besides, chromosomes 2 and 6 are also reported to have a large number of blast resistance genes (Figure 2). Another molecular marker-based approach allele mining was reported to clone and characterize many orthologues and alleles of blast *R* genes from wild rice [307,308,309,310,311]. Sheath blight considered the second most devastating fungal disease of rice which is caused by the necrotrophic fungus *Rhizoctonia solani* worldwide. Developing resistant cultivars against *R. solani* is very difficult due to the absence of a single major resistance gene, even though some resistance QTLs have been identified and used in rice improvement programs [312]. Brown spot caused by the necrotrophic fungus *Cochliobolus miyabeanus* (formerly known as *Helminthosporium oryzae*) is third in a row to damage rice crops by fungi. This fungus causes yield losses up to 52% [313]. For this fungus, only resistance QTLs have been detected [314]. Bakane disease does not have aneconomic importance as other major fungal diseases of rice, such as blast and sheath blight. It is caused by the *Gibberella fuzikori* species complex (GFSC). This complex comprises many species of *Fusarium fuzikori*, *F. proliferatum*, *F. concenticum* and *F. verticillioides*. The fungus causes infection through roots or crowns resulting in partially filled or chaffy grains. Only eight QTLs have been identified for the disease [270,315,316]. In addition, false smut caused by *Ustilaginoidea virens* is another minor fungal disease of rice. So far, except for tenQTLs, no major resistance-conferring gene has been identified from rice against this disease [286,317,318].

Several bacterial diseases also damage rice crops and major bacterial disease that cause significant yield losses in rice arediscussed here. A bacterial blight disease caused by the biotrophic bacterium *Xanthomonas oryzae* pv. *oryzae* (*Xoo*) is the utmost significant bacterial disease of rice, especially in tropical and subtropical areas. So far, more than 40 major resistance genes and several QTLs have been identified in rice using the marker-assisted selection method [319].Another bacterial disease, bacterial streak is caused by *X. oryzae* pv. *oryzicola* (*Xoc*) and it is a serious problem in Asian rice cultivation [320]. In this case, one *Xo1* locus in rice has been found to confer complete resistance to *Xoc* [321]. Numerous QTLs showing resistance to *Xoc* have been registered [319]. Two bacterial diseases, bacterial grain rot and bacterial seedling rot, produced by a necrotrophic bacterium called *Burkholderia glumae*, are important diseases of rice at the global level [322]. No single major resistance gene against *B. glumae* has been identified yet, but resistance QTLs have been reported through marker-assisted selection [314].

**Figure 3:**
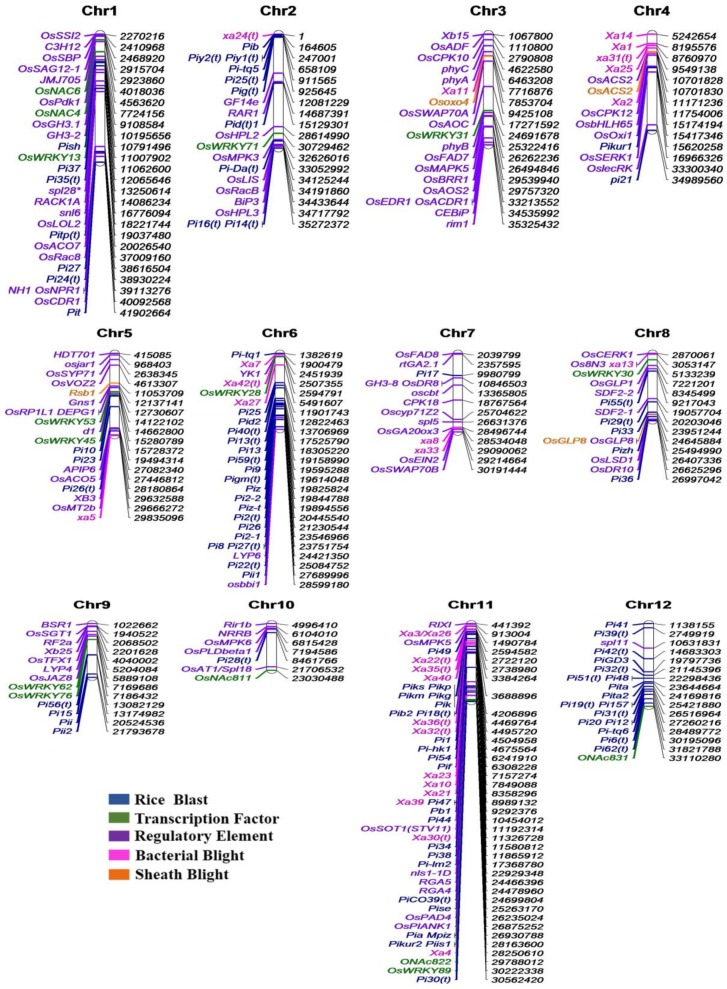
Distribution of major resistance genes according to theirphysical location on the respective chromosomes. Different disease resistance gene categories plotted on the chromosomes are indicated by five color codes. The plot was generated on the basis of the nearest linked molecular makers.

**Figure 4:**
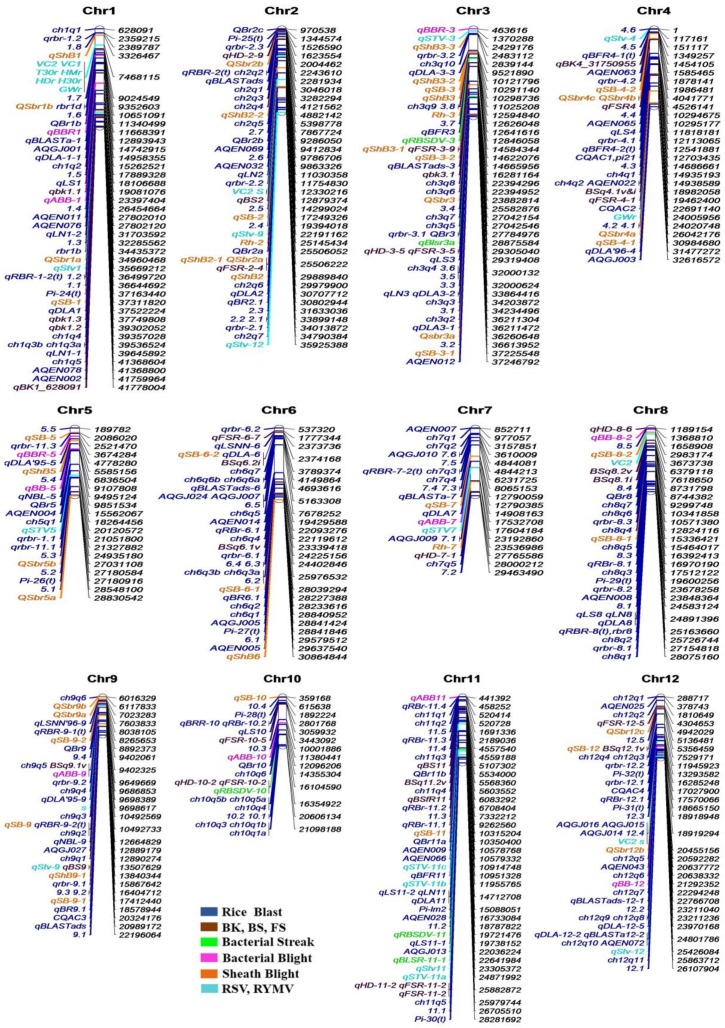
QTLs distribution on rice chromosomes. Separate color codes are given for each group of QTLs. The physical distribution of QTLs is derived by the nearest linked molecular markers on each chromosome. BK, BS, FS, RSV and RYMV represent Bacterial Streak, Brown Spot, False Smut, *Ricestripe virus* and *Rice yellow mottle virus* resistance QTLs, respectively.

Many viral diseases havealso been reported in rice and some of them are mentioned here, like rice stripe disease [323], rice yellow mottle disease [324,325] and rice tungro disease [326]. Rice stripe disease iscausedby the infection of *Rice stripe virus* which is an RNA virus transmitted by small vector brown plant hoppers. Five major resistance QTLs have been reported with the help of molecular marker techniques, and one of them wasmolecularly characterized at the nucleotide sequence level [327]. Rice tungro disease (RTD) consists of a *Rice tungro spherical virus* (RTSV) and a *Rice tungro* bacilliform virus (RTBV) and the disease is one of the most destructive diseases that causes yield constraint in rice-growing areas of tropical Asia. Both RTSV and RTBV are transferred in the host plant by an insect vector green leaf hopper (GLH) [328]. RTSV is freely transmitted by GLH, while RTBV can be transferred by GLH only in the presenceof helper virus RTSV [329]. Marker-assisted selection for resistance against RTSV has been effectively applied to develop RTSV-resistant rice lines [330]. Most of the major resistance genes and QTLs are plotted by chromosome-wise and remaining genes and QTLs could not be positioned on the respective chromosomes due to lack of their physical location (base pair) information (Figure 3 and Figure 4). Chromosomal distribution analysis showed that chromosomes 1, 6 and 11 had greater numbers of major genes and QTLs than other chromosomes, whereas considering only QTLs 2/3 rice chromosomes shared more or less equal numbers of loci (Figure 3). Chromosomes 4, 5, 7 and 10 contained less QTLs for disease resistance in rice (Figure 4).

### 7.2. Resistance Gene Pyramiding by MAS

Pyramiding is combining of genes into a single cultivar or line using back cross breeding. On the other hand, it is a strategy to combine two or more than two genes present in multiple parents into a single genotype with all of the target genes. Gene pyramiding is widely used for combining multiple pest or disease resistance genes for specific races of an insect or pathogen to generate durable resistance. By applying marker-assisted selection (MAS) and molecular marker back cross breeding (MMBC), it reduces the breeding duration in gene pyramiding and helps in crop improvement program. Different *R* genes often provides resistance to a number of different biotypes, races or isolates, thus making it durable. Several studies related to gene pyramiding have been reported in rice for disease resistance [331,332,333,334,335,336]. Recently, a group of four QTLs was pyramided in a rice line, giving resistance against a population of diverse *M. oryzae* isolates in different field conditions [337]. Similarly, many bacterial blight resistance genes have been pyramided and have developed improved rice cultivars using marker-assisted selection [338].

### 7.3. Genome-Wide Association Study of Resistance Genes

Association mapping is an alternative QTL mapping approach, conducting association analysis of genotypes versus phenotypes of a large population; it is based on linkage disequilibrium (LD) or the non-independence of alleles. In this method, SSR and SNP markers have been widely used to effectively identify marker and disease resistance associations in rice [339,340,341].Genome-wide association studies (GWASs) reflect association relationships of genome-wide distributed marker traits; such studies have become gradually popular in rice genetics with the advancement of high-throughput next generation sequencing (NGS) approaches and SNP chip techniques [342,343].GWAS is a very potent strategy which can be used for the understanding of the genetic basis of complex traits that has been exclusively useful for rice [332]. Nowadays, GWAS has been used in combination with NGS like genotyping by sequencing (GBS) to identify the SNP markers associated with resistance phenotypes in rice. Recently, an association study was performed based on 184 000 SNPs generated by GBS which helped in associating25 genomic regions with blast resistance in the rice genome [344].

### 7.4. Mutation Breeding for Rice Resistance

Mutation breeding is also known as the reverse genetics approach, in which mutants are generatedusing physical or chemical mutagens and evaluated for their resistance to various diseases. Most of the mutants generated by this method are deleterious in nature, but very few show promising effects for agronomically important traits including disease resistance. This approach is very useful in those crops which do not have much genetic diversity, but it has been routinely used in rice for disease resistance [345,346,347]. Various mutagenic agents like γ, UV, X-rays irradiation (Physical mutagens), ethyl methane sulfonate (EMS), methyl methane sulfonate (MMS) and colchicine, (Chemical mutagens) are generally used for the induction of mutations. However, biological mutagens like T-DNA insertion and transposable elements have been widely exploited by plant breeders to generate mutant lines [348]. The main drawbacks of mutation breeding arethe restricted influence in producing dominant alleles with less efficiency and also its random nature.

## 8. Transgenic Approaches for Disease Resistance in Rice

With the recent advances in sequencing technologies, various genes involved in pathogenesis pathways and plant innate immunity have been dissected and used for developing durable disease-resistant crops through transgenic approaches like over-expression/gene complementation tests, Small RNA (microRNA), RNA interference (RNAi), CRISPR/Cas systems [349]. However, genetically modified (GM) plants derived usingthese approaches have been adapted in the main stream of agriculture at a very small scale. Due to various potential biosafety issues, introduction of new allergens into food, horizontal gene transfer from GM plants to non GM plants or microbes, genetic diversity loss, affecting non target organisms, and socioeconomic and ethical concerns [350,351,352,353], involved with the transgenic approaches. Although there is no doubt that the GM technology has played an important role to fulfil our increased demands in the field of medicine and some non-food crop plants, plant molecular biology and biotechnology techniques have taken a rapid progress in the identification and cloning of genes involved in plant defense responses. In the following sections, various transgenic approaches being used for the development of disease resistance in rice are explained.

### 8.1. Over-Expression/Functional Complementation Test in Rice Resistance

The term “overexpression” means increased expression of targeted genes beyond the normal expression level. The functional complementation assay (FCA) is an in vivo assay commonly used to validate gene function and its essentiality. For biotic stresses, the number of genes has already been characterized using the complementation test. For developing resistance, several blast resistance (*R*) genes from rice have already been identified and characterized [1,311,354].Now, many *R* genes have been fully characterized in rice and their effectiveness has beentested using various approaches (Table 3). The resistance spectrum of *R* gene orthologues by mining superior alleles from wild species with a broader defense spectrum has also been reported [307,311].

Due to continuous selection pressure, pathogens overcome *R*-mediated host resistance in a few years [395]. To overcome such a scenario, gene stacking methods are used, i.e., combing two or more genes for developing crops with combined protection against several pathogens. Recently, two *R* gene alleles, i.e., *Pi54* and *Pi54rh* were stacked together in a susceptible rice variety to confer enhanced combined host resistance against *M. oryzae* [362,396].Similarly, transgenic rice expressing two fusion genes *mpi* and *pci* (proteinase inhibitors) has displayed high resistance towards insect attack and rice blast infection [363]. Richa et al. [369] also developed transgenic rice plants harboring novel *chitinase* gene (LOC_Os11g47510) through genetic transformation resulting in much higher resistance against sheath blight (ShB) disease. Over-expressing thaumatin-like protein (TLP) in a rice line showed an enhanced level of ShB resistance compared to the control plants [370].Using a gene stacking method, chitinase gene (*RCH10*) and glucanase gene (*AGLU1*) together provide resistance to both ShB and rice blast pathogen in a susceptible rice variety [371]. SA, JA and ET plant hormones play key roles in defense responses and signaling [397,398]; they are also known to activate many defense-associated kinases, different transcription factors and various *PR* genes, as these genes have been reported to increase the host resistance in many transgenic rice lines [399]. Transgenic rice expressing *OsACS2* gene encoded 1-aminocyclopropane-1-carboxylic acid synthase, which is a key enzyme of ET biosynthesis, under the regulation of a pathogen-inducible promoter resulted in higher ET production and enhanced resistance against rice blast and sheath blight diseases [367]. In a similar study, *MoSM1*-overexpressing transgenic rice showed an improved resistance against *M. oryzae*, and *Xoo* by modulating SA/JA signaling pathways [389]. Interestingly, some reports mentioned increasing host resistance because of protein elicitors produced by plant pathogens. Two elicitors, namely, *MoHrip1* and *MoHrip2* cloned from *M. oryzae* fungus, when overexpressed in rice result in lower levels of disease severity compared to the controls [388]. Small RNAs including siRNAs (short-interfering RNAs) and miRNAs (microRNAs) are the major groups associated with post-transcriptional gene regulation affecting eukaryotic immunity. These are the short non-coding RNAs having a consistentmode of biogenesis and mechanism of action. With the advancement in sequencing technology, large sets of miRNAs have been identified and characterized in various crops for both biotic and abiotic stresses. Using deep sequencing, Li et al. [374] explored miRNA for rice immunity against *M. oryzae*. Transgenic rice plants with over-expressing miR160a and miR398b resulted in up-regulated defense-related genes followed by enhanced resistance against *M. oryzae*. Similarly, Campo et al. [376] identified and overexpressed novel osa-miR7695 miRNA in rice. Transgenic plants harboring osa-miR7695 have ahigh resistance spectrum against the rice blast pathogen; miR169a overexpressing rice lines were found to be highly affected by *M. oryzae* infection by altering defense related responses [375]. Thus, the miRNA acts in both ways as a positive- as well as negative-regulator towards plant immunity.

### 8.2. Role of RNA Interference (RNAi) in Disease Resistance

The RNA interference (RNAi) approach involves sequence-specific gene regulation mediated by small RNAs (sRNAs). In eukaryotes, RNAi emerged as one of the most precise, efficient mechanism resulting in gene regulation both at the transcriptional and post transcriptional level. RNAi plays critical roles in developmental regulation, stress response, and host defense against transposons and viruses. It involves a two-step mechanism, wherein its initial step is to degrade dsRNA and thereby generates 21–25 nucleotides long small interfering RNAs (siRNAs) through the action of RNase III-like molecules. In the final step, siRNAs associate with an RNase and make RNA-induced silencing complex (RISC), which precisely acts on the cognate partner of double-stranded mRNA and ultimately degrade the targeted mRNAs via a homology dependent manner [400,401,402].Various target traits have already been modified through RNAi approaches towards crop improvement (Table 3). RNA-dependent RNA polymerases (RDR) play a crucial role in gene silencing that provide resistance against the pathogens. Wagh et al. [403] reported the mutantrice line of *RDRP6* gene increased susceptibility response against *Cucumber mosaic virus* (CMV), *Rice necrosis mosaic virus* (RNMV), *X. oryzae* pv. *oryzae* and *M. oryzae*. Host-induced gene silencing (HIGS) is a mechanism that involves the silencing of pathogen genes using the RNAi tool [404]. The target genes of pathogens are expressed and dsRNA is generated using the plant machinery. This dsRNA is used as a precursor for generating smaller RNA fragments complementary to the genes expressed distantly in the pathogen [405]. There are transgenic rice lines carrying a hybrid RNAi construct targeting two pathogen genes where pathogenicity MAP kinases *RPMK1-1* and *RPMK1-2* shows increased sheath blight resistance compared to the control lines [390]. VIGS (Virus Induced Gene Silencing) is an important tool for triggering RNAi silencing with the use of viral vectors like BMV (*Brome mosaic virus*). VIGS acts as an efficient and rapid tool for assigning gene function in plants. Using BMV-HIGS, Zhu et al. [406] reported that *MoABC1*, *MoMAC1* and *MoPMK1 M. oryzae* genes were responsible for disease development. 

### 8.3. CRISPR/Cas9 Immune System

With sequence-specific nucleases (SSNs), genome editing where gene insertion, deletion or replacement in the genome become a reality with enormous possibilities. SSNs are also considered as “molecular scissors” which belong to four categories, i.e., MegaN (mega nuclease), ZFNs (Zinc finger nucleases), TALENs (transcription activator-like effector nucleases) and CRISPR/Cas9 (clustered regularly interspaced short palindromic repeat/CRISPRs-associated protein 9) [407,408]. CRISPR/Cas9 system is considered as one of the most important and simple genome editing tool. Due to high simplicity and efficiency, this system becomes a powerful tool for understanding various biosynthetic pathways and resistance response mechanisms in crop plants [392]. This system is not an exception of off-target problems associated with other genome editing tools ZFNs and TALENs. In this system, the off-target percentage is usually insignificant and it can be further diminished by considering a few points like designing specific guide RNA (sgRNA) sequences, complete whole genome sequence information of the experimental crop plant and selection of highly precise computational tools for target identification [409,410,411]. Rice as a diploid and a monocot plant is considered one of the best choices for the CRISPR/Cas9 system. The *OsSWEET14* gene in rice results in the pathogenesis of *X.oryzae*. Modifying effector-binding sites present in the *OsSWEET14* gene promoter results in reduction ofpathogen virulence [393]. For addressing biotic stress resistance in rice using CRISPR/Cas9, Wang et al. [394] developed targeted knockout of the *OsERF922* gene and achieved improved rice blast resistance without affecting agronomic traits.

## 9. Conclusions and Future Perspectives

Rice wasthe firstcrop plant to be decoded at the genome level, and its sequence information has been publicly available since 2005. Thereafter, this crop has attracted lots of attention from plant molecular biologists in relation to study the disease resistance and other agronomically important traits.Although substantial advances have been attained regardinginsights into the genetic nature of disease resistance genes and signal transduction pathways along with influencing regulatory factors heading to defense response activation in rice, the complete story is still far from well-defined. Rice is a well-known model crop plant for various research activities, however, it has lesser information than other model plants like *Arabidopsis* and tobacco with reference to disease resistance. Host resistance response can be very effectively improved by using modern molecular biology and genetic engineering techniques. However, such resistance responsesaregenerally broken down by the emergence of more virulent races of the pathogen. By characterizing additional *R* genes from rice [238,262] wild species and local rice landraces, plant genotypes with varying degree of disease resistance can be obtained [412]. Understanding the signaling cascades involved in disease resistance and the host defense pathway-associated genes can be achieved by the application of the latest molecular biology approaches. These signaling genes will be very helpful for developing rice varieties with sustainable and broad spectrum resistance against various pathogens. Thus, long-term durable and broad spectrum resistance rice is our current need in view of global climatic changes which may help in the emergence of new and virulent races of the pathogens. This goal of getting broad spectrum resistance can be achieved through several emerging approaches like hostplant immunity, non-host resistance, multigene varieties, interspecific gene transfer and genome editing, etc.

Plants possess PTI and ETI innate immune systems to withstand different biotic stresses. While, pathogens are equipped with advanced effector molecules that defeat host plant immunity, many importantconclusions have been drawn regardingthe rice–pathogen interactions using different techniques such as plant breeding, mutation breeding, marker-assisted selection, gene pyramiding, association studies, genetic engineering with complementation tests, RNAi, miRNA, CRISPR/Cas9, etc. Several important outcomes related to disease resistance have been discovered, such as hypersensitive response (HR) by major *R* gene, ROS generation, *PR* gene activation, hormone biosynthesis and their cross talk with other signaling pathways. However, the entire connections and factors engaged in the plant immune responses employing disease resistance genes are still not clear in rice. The information provided in this review will help in the understanding ofdifferent rice hostpathosystems that may lead to the development of sustainable broad spectrum disease-resistant cultivars.

## Figures and Tables

**Table 1:** Details of the resistance genes identified from rice.

Chromosome	Genes	References
1	*(Xa29(t))*, *(Pit*, *Pitp(t)*, *Pi37*, *Pi35(t)*, *Pi24(t)*, *Pi27*, *Pish)*	[136,149,150,151,152,153,154,155]
2	*(xa24(t))*, *(Pi*-*b*, *Pi25(t)) (Pid1(t)*, *Pi*-*Da(t))*, *(Pi*-*y1(t)*, *Piy2(t))*, *(Pig(t)*, *Pi*-*tq5*, *Pi14(t)*, *Pi16(t))*	[149,153,156,157,158,159,160,161,162,163]
3	*(Xa11)*, *(xa42)*, *(Pi66(t))*	[164,165,166,167]
4	*(Xa1)*, *(Xa2)*, *(Xa12)*, *(Xa14)*, *(xa31(t))*, *(Pi39(t)*, *pi21*, *Pikur1*, *Pi(t))*	[58,168,169,170,171,172,173,174,175,176,177,178]
5	*(xa5)*, *(Pi26(t)*, *Pi23*, *Pi10)*	[153,179,180,181,182]
6	*(Xa7)*, *(Xa27)*, *(xa33(t))*, *(Pi27(t)*, *Pi*-*tq1*, *Pi8*, *Pi13(t)*, *Pi22(t)*, *Pigm(t))*, *(Piz*-*5*, *Piz*-*t)*, *(Pi40(t)*, *Pi59(t)*, *Pi9*, *Pi2*-*1*, *Pid2)*, *(Pi25(t)*, *Pi26)*,(*Piz)*, *(Pi13)*, *(Pi2*-*2*, *Pi50(t))*	[153,161,162,163,181,183,184,185,186,187,188,189,190,191,192,193,194,195,196,197,198,199,200,201]
7	*(xa8)*, *(Pi17(t))*	[202,203]
8	*(xa13), (Pi29(t), Pi33, Pizh, Pi36, pi55(t), PiGD-1(t))*	[153,204,205,206,207,208,209,210,211,212,213]
9	*(Pi15*, *Pi2(t)*, *Pi3(t)*, *Pi5(t)*, *Pi56(t))*	[214,215,216,217,218]
10	*(Pi28(t)*, *PiGD*-*2(t))*	[153,213]
11	*(Xa3/Xa26), (Xa4), (Xa6, xa9), (Xa10), (Xa21), (Xa22), (Xa23, Xa30(t), Xa32(t), Xa35(t), Xa39, Xa40(t)), (Pi7(t)), (Pik, Pik-p), (Pi30(t), Pi60(t), Pilm2, Pikg),(Pik-h, Pik-s), (Pi-hk1), (Pi54), (Pi-1(t), (Pb1, Pise1, Pikur2, Pi38, Pif, Pi34, Pia, PiCO39(t), Pi44(t), Pi49, Pik-m, Pi18(t), Pi47), (Pi1)*	[129,149,153,159,161,162,183,197,219,220,221,222,223,224,225,226,227,228,229,230,231,232,233,234,235,236,237,238,239,240,241,242,243,244,245,246,247,248,249,250,251,252,253,254]
12	*(xa25/Xa25(t)), (Pita-2), (Pi31(t), Pi32(t)), Pi61(t), Pi-tq6, (Ipi(t), IPi3(t)), (Pi21(t), Pi58(t), Pi51(t), Pi-GD-3(t), Pi48,Pi24(t), Pi-42(t), Pi62(t)), (Pi6(t), Pi4(t)), (Pi12(t), Pi19(t), Pita, Pi39(t), Pi20(t))*	[149,153,159,161,178,181,192,194,213,252,255,256,257,258,259,260,261,262,263,264]

*Xa* indicates resistance gene to bacterial blight disease (*Xoo*) and *Pi* represents resistance gene against blast disease (*M. oryzae*).

**Table 2:** Chromosome wise list of identified QTLs against various pathogen induced diseases in rice.

Chromosome	QTLs	References
1	*(1.1*, *1.2*, *1.3*, *1.4*, *1.5*, *1.6*, *1.7*, *1.8)*,*(AQEN002*, *AQEN011*, *AQEN076*, *AQEN078)*, *AQGJ001*,( *ch1q1*, *ch1q2*, *ch1q3a*, *ch1q3b*, *h1q4*, *ch1q5)*,*(GWr*, *H30r*, *HDr*, *HMr, T30r*, *VC1*,*VC2)*, *Pi*-*24(t)*, *qABB*-*1*, *qBBR1*,*(qbk1.1*, *qbk1.2*, *qbk1.3)*, *qBK1_628091*, *qBLASTa*-*1*, *qBlsr1*,*(QBr1a*, *QBr1b)*, *qBSfR1*,*(qDLA1, qLN1*-*1*, *qLN1*-*2*, *qLS1)*, *qDLA*-*1*-*1*, *qFSR1*, *qrbr*-*1.2*,*(qRBR*-*1*-*1(t)*, *rbr1a*, *qRBR*-*1*-*2(t)*, *rbr1b*, *qRBR*-*1*-*4(t)*, *rbr1d)*, *qSB*-*1*,*(QSbr1a*, *QSbr1b)*, *qShB1*, *qStv1*,	[129,153,196,199,265,266,267,268,269,270,271,272,273,274,275,276,277,278,279,280,281,282,283]
2	*(2.1*, *2.2*, *2.3*, *2.4*, *2.5*, *2.6*, *2.7)*,*(AQEN032*, *AQEN069)*,*(BSq2.1v&i*, *BSq2.2v&i)*,*(ch2q1*, *ch2q2*, *ch2q3*, *ch2q4*, *ch2q5*, *ch2q6*, *ch2q7)*, *Pi*-*25(t)*, *qBLASTads*, *qBlsr2*, *qBR2.1*,*(QBr2a*, *QBr2b*, *QBr2c)*, *qBS2*,*(qDLA2, qLN2)*, *qFSR*-*2*-*4*,,*(qRBR*-*2(t)*, *rbr2)*,*(qrbr*-*2.2*, *qrbr*-*2.3)*, *qSB*-*2*,*(QSbr2a*, *QSbr2b), qShB2*,*(qShB2*-*1*, *qShB2*-*2)*,*(qStv*-*12*,*qStv*-*9)*, *Rh*-*2*,*(S*, *VC2)*	[129,153,196,199,266,267,272,273,274,278,280,281,282,284,285,286,287,288,289,290,291,292]
3	*(3.1*, *3.2*, *3.3*, *3.4*, *3.5*, *3.6*, *3.7*, *3.8*, *qFSR*-*3*-*5*, *qFSR*-*3*-*9)*, *AQEN012*,*(ch3q10*, *ch3q2*, *ch3q3*, *ch3q4*, *ch3q5*, *ch3q6*, *ch3q7*, *ch3q8*, *ch3q9)*, *qBBR*-*3*, *qBBR3*-*1*, *qBFR3*, *qbk3.1*, *qBLASTads*-*3*,*(qBlsr3a*, *qBlsr3b*, *qBlsr3c*, *qBlsr3d)*, *QBr3*,*(qDLA3*-*1*, *DLA3*-*2, qLN3*, *qLS3)*, *qDLA*-*3*-*3* ,*(qrbr*-*3.1*, *qrbr*-*3.2)*, *qRBSDV*-*3*, *qSB*-*3*,*(qSB*-*3*-*1*, *qSB*-*3*-*2)*, *QSbr3*, *Qsbr3a*, *qShB3*,*(qShB3*-*1*, *qShB3*-*2*, *qShB3*-*3)*, *qSTV*-*3*, *Qxa*-*3*, *Rh*-*3*	[129,196,199,266,269,270,272,273,274,276,278,280,281,282,288,290,292,293,294,295,296,297,298]
4	*(4.1*, *4.2*, *4.3*, *4.4*, *4.5*, *4.6)*, *qFSR*-*4*-*1*,( *AQEN022*, *AQEN063*, *AQEN065)*, *AQGJ003*, *BSq4.1v&i*,*(ch4q1*, *ch4q2)*,*(CQAC1*, *pi21*, *CQAC2)*, *GWr*, *qBB*-*4*, *qBBR*-*4*,*(qBFR4*-*1(t)*, *qBFR4*-*2(t))*, *qBK4_31750955*,*(qBlsr4a*, *qBlsr4b)*, *QBr4*, *qBSfR4*, *qDLA’96*-*4*, *qFSR4*, *qLS4*,*(qrbr*-*4.1*, *qrbr*-*4.2)*,*(qSB*-*4*-*1*, *qSB*-*4*-*2)*,*(QSbr4a*, *QSbr4b*, *QSbr4c)*, *qStv*-*4*	[129,196,199,265,266,267,268,271,273,274,275,276,277,278,280,281,284,286,291,293,299,300]
5	*(5.1*, *5.2*, *5.3*, *5.4*, *5.5)*, *AQEN004*, *ch5q1*, *Pi*-*26(t)*, *qBB*-*5*, *qBBR*-*5*,*(qBlsr5a*, *qBlsr5b)*, *QBr5*,*(qDLA’95*-*5*, *qNBL*-*5)*,*(qrbr*-*1.1*, *qrbr*-*11.1*, *qrbr*-*11.3*, *qrbr*-*2.1)*, *qSB*-*5*,*(QSbr5a*, *QSbr5b)*, *qShB5*, *qSTV5*, *xa5*	[129,153,199,266,268,273,274,276,278,281,282,290,293,298,301,302]
6	*(6.1*, *6.2*, *6.3*, *6.4*, *6.5)*, *qFSR*-*6*-*7*,*(AQEN005*, *AQEN014)*,*(AQGJ005*, *AQGJ007*, *AQGJ024)*,*(BSq6.1v*, *BSq6.2i)*,*(ch6q1*, *ch6q2*, *ch6q3a*, *ch6q3b*, *ch6q4*, *ch6q5*, *ch6q6a*, *ch6q6b*, *ch6q7)*, *Pi*-*27(t)*, *qBBR*-*6*,*(qBLASTads*-*6*, *Pi*-*tq1)*, *qBR6.1*,*(qDLA*-*6*, *qLSNN*-*6)*,*(qrbr*-*6.1*, *qrbr*-*6.2)*,*(qSB*-*6*-*1*, *qSB*-*6*-*2)*, *qShB6*, *xa7*	[129,153,199,265,266,272,276,278,280,282,284,286,293,298,303]
7	*(7.1*, *7.2*, *7.3*, *7.4*, *7.5*, *7.6)*, *AQEN007*,*(AQGJ009*, *AQGJ010)*,*(ch7q1*, *ch7q2*, *ch7q3*, *ch7q4*, *ch7q5)*, *qABB*-*7*, *qBBR7*, *qBLASTa*-*7*, *QBr7*, *qDLA7*,*(qRBR*-*7*-*2(t)*, *rbr7b)*, *qSB*-*7*, *qStv7*, *Rh*-*7*	[129,196,199,265,266,268,269,272,274,279,288,292,302]
8	*(8.1*, *8.2*, *8.3*, *8.4*, *8.5)*, *qFSR*-*8*-*3*, *AQEN008*,*(BSq8.1i*, *BSq8.2v)*,*(ch8q1*, *ch8q2*, *ch8q3*, *ch8q4*, *ch8q5*, *ch8q6*, *ch8q7)*, *Pi*-*29(t)*,*qBB*-*8*-*2*, *QBr8*,*(qDLA8*, *qLN8*, *qLS8)*,*(qRBR*-*8(t)*, *rbr8)*,*(qrbr*-*8.1*, *qrbr*-*8.2*, *qrbr*-*8.3)*,*(qSB*-*8*-*1*, *qSB*-*8*-*2)*, *Qsbr8a*, *VC2*	[129,153,196,199,266,267,268,274,278,279,280,284,286,296]
9	*(9.1*, *9.2*, *9.3*, *9.4)*, *AQGJ027*, *BSq9.1v*,*(ch9q1*, *ch9q2*, *ch9q3*, *ch9q4*, *ch9q5*, *ch9q6)*, *CQAC3*, *qABB*-*9*, *qBLASTads*, *QBr9*, *qBR9.1*, *qBS9*,*(qDLA’95*-*9*, *qLSNN’96*-*9*, *qNBL*-*9)*,*(qrbr*-*9.1*, *qrbr*-*9.2)*,*(qRBR*-*9*-*1(t)*, *rbr9a*, *qRBR*-*9*-*2(t)*, *rbr9b)*, *qSB*-*9*,*(qSB*-*9*-*1*, *qSB*-*9*-*2)*,*(QSbr9a*,*QSbr9b)*, *qShB9*-*1*, *qStv*-*9*, *s*	[199,213,265,266,267,268,272,274,276,278,279,280,281,282,284,285,289,291,299,301]
10	*(10.1*, *10.2*, *10.3*, *10.4)*,*(qFSR*-*10*-*2*, *qFSR*-*10*-*5)*,*(ch10q1a*, *ch10q1b*, *ch10q2*, *ch10q3*, *ch10q4*, *ch10q5a*, *ch10q5b*, *ch10q6)*, *Pi*-*28(t)*, *qABB*-*10*, *QBr10*, *qFSR10*, *qLS10*, *qRBSDV*-*10*, *qSB*-*10*	[153,196,199,266,268,277,280,281,286,295]
11	*(11.1*, *11.2*, *11.3*, *11.4*, *11.5)*, *qFSR*-*11*-*2*,*(AQEN009*, *AQEN028*, *AQEN066)*, *AQGJ013*,*(BSq11.1v&i*, *BSq11.2v)*,*(ch11q1*, *ch11q2*, *ch11q3*, *ch11q4*, *ch11q5)*, *Pi*-*30(t)*, *Pi*-*lm2*, *qABB11*, *qBFR11*, *qBlsr11*, *QBr11a*, *QBr11b*, *qBS11*, *qBSfR11*,*(qDLA11*, *qLN11*, *qLS11*-*1*, *qLS11*-*2)*, *qRBSDV*-*11*, *qSB*-*11*, *qStv11*,*(qSTV*-*11a*, *qSTV*-*11b*, *qSTV*-*11c)*	[129,153,196,199,265,266,268,274,277,281,284,286,294,297,304,305]
12	*(12.1*, *12.2*, *12.3*, *12.4*, *12.5)*, *qFSR*-*12*-*5*,*(AQEN025*, *AQEN043*, *AQEN072)*,*(AQGJ014*, *AQGJ015*, *AQGJ016)*, *BSq12.1v*,*(ch12q1*,, *ch12q2*, *ch12q3*, *ch12q4*, *ch12q5*, *ch12q6*, *ch12q7*, *ch12q8*, *ch12q9, ch12q10*, *ch12q11)*, *CQAC4*,*(Pi*-*31(t)*, *Pi*-*32(t))*, *qBB*-*12*,*(qBLASTa12*-*2*, *qBLASTads*-*12*-*1*, *Pi*-*tq6)*, *(qDLA*-*12*-*2*, *qDLA*-*12*-*5)*, *qFSR12*,*(qrbr*-*12.1*, *qrbr*-*12.2)*, *qSB*-*12*,*(QSbr12a*, *QSbr12b*, *QSbr12c)*, *qStv*-*12*,*(s*, *VC2)*	[129,153,199,265,266,267,268,272,276,277,278,280,281,284,286,291,299]

**Table 3:** List of selected rice resistance and defense response genes characterized by genetic engineering.

Gene	Type	Method	Pathogen	Reference
*Pib*	NBS-LRR	OE	*M. oryzae*	[355]
*Pi*-*ta*	NBS-LRR	OE	*M. oryzae*	[262]
*Pi9*	NBS-LRR	OE	*M. oryzae*	[193]
*Pi-2*	NBS-LRR	OE	*M. oryzae*	[190]
*Pi5*	CC-NB-LRR	OE	*M. oryzae*	[356]
*Pi21*	Proline-rich protein	RNAi	*M. oryzae*	[357]
*Pi36*	CC-NB-LRR	OE	*M. oryzae*	[263]
*Pi37*	NBS-LRR	OE	*M. oryzae*	[151]
*Pi54*	NBS-LRR	OE	*M. oryzae*	[358]
*pi-d2*	B-lectin domain	OE	*M. oryzae*	[195]
*Pik*	CC-NBS-LRR	OE & RNAi	*M. oryzae*	[359]
*Pikm*	NBS-LRR	OE	*M. oryzae*	[360]
*Pik-p*	CC-NBS-LRR	OE & RNAi	*M. oryzae*	[361]
*Pi54rh*	NBS-LRR	OE	*M. oryzae*	[307]
*Pi54of*	CC-LRR	OE	*M. oryzae*	[311]
*Pi54* and *Pi54rh*	NBS-LRR	OE	*M. oryzae*	[362]
*mpi* and *pci*	Proteinase inhibitors	OE	*M. oryzae*& Insect pests	[363]
*chi11* and *ap24*	Rice chitinase & Tobacco osmotin	OE	*R. solani*	[364]
*OsBRR1*	LRR-RLK	OE	*M. oryzae*	[112]
*OsWAK1*	Protein kinase	OE	*M. oryzae*	[365]
*Dm-AMP1*	Antifungal plant defensin	OE	*M. oryzae*&*R. solani*	[366]
*OsACS2*	Ethylene biosynthetic gene	OE	*M. oryzae*&*R. solani*	[367]
*At. NPR1*	Defense gene	OE	*R. solani*	[368]
(Loc_Os11g47510)	Chitinase	OE	*R. solani*	[369]
*PR*-*5*	Thaumatin-like protein	OE	*R. solani*	[370]
*RCH10* and *AGLU1*	Chitinase & Alfalfa β-1,3-glucanase gene	OE	*M. oryzae*&*R. solani*	[371]
*OsCPK4*	Protein kinase	OE	*M. oryzae*&*R. solani*	[372]
*OsCPK10*	Protein kinase	OE	*M. oryzae*&*R. solani*	[373]
*miR160a* and *miR398b*	miRNA	OE	*M. oryzae*	[374]
*miR169a*	miRNA	OE	*M. oryzae*	[375]
*osa*-*miR7695*	miRNA	OE	*M. oryzae*	[376]
*OsPGIP1*	Polygalacturonase inhibiting proteins	OE	*R. solani*	[377]
*BSR1*	Receptor like kinase	OE	*Xoo*, *M. oryzae, Burkholderiaglumae, Cochliobolusmiyabeans*, RSV	[378]
*OsOSM1*	Osmotin protein (PR)	OE	*R. solani*	[379]
*OsPGIP1*	Polygalacturonase inhibiting proteins	OE	*R. solani*	[380]
*OsPR1b*	PR	OE	*Xoo*	[381]
*OsSAMS1*	*S*-adenosyl-l-methionine synthetase	RNAi	RDV	[382]
*OsDCL1*	Dicer-like	RNAi	*M. oryzae*	[383]
*miR528*	miRNA	OE	RDV	[384]
*miR168*	miRNA	RNAi	RDV, RSV	[385]
*ALS*	Acetolactate Synthase	CRISPR/Cas9	Herbicide	[386]
*OsSWEET13*	Sucrose transport	TALENs	*Xoo*	[387]
*MoHrip1 and MoHrip2*	Effector protein	OE	*M. oryzae*	[388]
*MoSM1*	Secreted protein	OE	*M. oryzae*&*Xoo*	[389]
*RPMK1*-*1 and RPMK1-2*	Kinases	RNAi	*R. solani*	[390]
*chi11*	Chitinase	OE	*R. solani*	[391]
*OsSWEET11, OsSWEET14*	Sucrose transporter	CRISPR/Cas9	*Xoo*	[392]
*OsSWEET14*	Sucrose transporter	TALEN	*Xoo*	[393]
*OsERF922*	ERF	CRISPR/Cas9	*M. oryzae*	[394]

Over-expression: OE, Rice sheath blight pathogen: *R. solani*, *Rice stripe virus*: RSV, *Rice dwarf virus*: RDV, Ethylene Responsive Factor: ERF.

## References

[1] Sharma T.R., Rai A.K., Gupta S.K., Vijayan J., Devanna B.N., Ray S. (2012). Rice blast management through host-plant resistance, retrospect and prospects. Agric. Res..

[2] Khush G.S. (2005). What it will take to feed 5.0 billion rice consumers in 2030. Plant Mol. Biol..

[3] Das G., Rao G.J. (2015). Molecular marker assisted gene stacking for biotic and abiotic stress resistance genes in an elite rice cultivar. Front. Plant Sci..

[4] Ou S.H. (1985). Rice Diseases.

[5] Jones J.D., Dangl J.L. (2006). The plant immune system. Nature.

[6] Bray Speth E., Lee Y.N., He S.Y. (2006). Pathogen virulence factors as molecular probes of basic plant cellular functions. Curr. Opin. Plant Biol..

[7] Nurnberger T., Kemmerling B. (2009). Pathogen-associated molecular patterns (PAMP) and PAMP-triggered immunity. Annu. Plant Rev..

[8] Chen X., Ronald P.C. (2011). Innate immunity in rice. Trends Plant Sci..

[9] Dangl J.L., Jones J.D.G. (2001). Plant pathogens and integrated defence responses to infection. Nature.

[10] Ausubel F.M. (2005). Are innate immune signaling pathways in plants and animals conserved?. Nat. Immunol..

[11] Chisholm S.T., Coaker G., Day B., Staskawicz B.J. (2006). Host-microbe interactions: Shaping the evolution of the plant immune response. Cell.

[12] Zipfel C., Felix G. (2005). Plants and animals: A different taste for microbes?. Curr. Opin. Plant Biol..

[13] Freeman B.C., Beattie G.A. (2008). An overview of plant defenses against pathogens and herbivores. Plant Health Instruct..

[14] Serrano M., Coluccia F., Torres M.L., Haridon F., Metraux J.P. (2014). The cuticle and plant defense to pathogens. Front. Plant Sci..

[15] Doughari J. (2015). An overview of plant immunity. J. Plant Pathol. Microbiol..

[16] Nicaise V., Roux M., Zipfel C. (2009). Recent advances in PAMP-triggered immunity against bacteria: Pattern recognition receptors watch over and raise the alarm. Plant Physiol..

[17] Tena G., Boudsocq M., Sheen J. (2011). Protein kinase signaling networks in plant innate immunity. Curr. Opin. Plant Biol..

[18] Chassot C., Nawrath C., Metraux J. (2007). Cuticular defects lead to full immunity to a major plant pathogen. Plant J..

[19] Tonnessen B.W., Manosalva P., Lang J.M., Baraoidan M., Bordeos A., Mauleon R., Oard J., Hulbert S., Leung H., Leach J.E. (2015). Rice phenylalanine ammonia-lyase gene *OsPAL4* is associated with broad spectrum disease resistance. Plant Mol. Biol..

[20] Manosalva P.M., Davidson R.M., Liu B., Zhu X., Hulbert S.H., Leung H., Leach J.E. (2009). A germin-like protein gene family functions as a complex quantitative trait locus conferring broad-spectrum disease resistance in rice. Plant Physiol..

[21] Yamaguchi K., Yamada K., Ishikawa K., Yoshimura S., Hayashi N., Uchihashi K., Ishihama N., Kishi-Kaboshi M., Takahashi A., Tsuge S. (2013). A receptor-like cytoplasmic kinase targeted by a plant pathogen effector is directly phosphorylated by the chitin receptor and mediates rice immunity. Cell Host Microbe.

[22] Ao Y., Li Z., Feng D., Xiong F., Liu J., Li J., Wang M., Wang J., Liu B., Wang H. (2014). *OsCERK1* and *OsRLCK176* play important roles in peptidoglycan and chitin signaling in rice innate immunity. Plant J..

[23] Cayrol B., Delteil A., Gobbato E., Kroj T., Morel J.-B. (2016). Three wall-associated kinases required for rice basal immunity from protein complexes in the plasma membrane. Plant Signal. Behav..

[24] Li Z.Y., Xia J., Chen Z., Yu Y., Li Q.F., Zhang Y.C., Zhang J.P., Wang C.Y., Zhu X.Y., Zhang W. (2016). Large-scale rewiring of innate immunity circuitry and microRNA regulation during initial rice blast infection. Sci. Rep..

[25] Cheng X., Wu Y., Guo J., Du B., Chen R., Zhu L., He G. (2013). A rice lectin receptor like kinase that is involved in innate immune responses also contributes to seed germination. Plant J..

[26] Shiu S.-H., Bleecker A.B. (2001). Receptor-like kinases from *Arabidopsis* form a monophyletic gene family related to animal receptor kinases. Proc. Natl. Acad. Sci. USA.

[27] Kopp E., Medzhitov R. (2003). Recognition of microbial infection by Toll-like receptors. Curr. Opin. Immunol..

[28] Schwessinger B., Ronald P.C. (2012). Plant innate immunity: Perception of conserved microbial signatures. Annu. Rev. Plant Biol..

[29] Macho A.P., Zipfel C. (2014). Plant PRRs and the activation of innate immune signaling. Mol. Cell.

[30] Li L., Yu Y., Zhou Z., Zhou J.M. (2016). Plant pattern-recognition receptors controlling innate immunity. Sci. China Life Sci..

[31] Navarro L., Dunoyer P., Jay F., Arnold B., Dharmasiri N., Estelle M., Voinnet O., Jones J.D.G. (2006). A plant miRNA contributes to antibacterial resistance by repressing auxin signaling. Science.

[32] Pumplin N., Voinnet O. (2013). RNA silencing suppression by plant pathogens: Defence, counter-defence and counter-counter-defence. Nat. Rev. Microbiol..

[33] Katiyar-Agarwal S., Morgan R., Dahlbeck D., Borsani O., Villegas A., Zhu J.K., Staskawicz B.J., Jin H. (2006). A pathogen-inducible endogenous siRNA in plant immunity. Proc. Natl. Acad. Sci. USA.

[34] Staiger D., Korneli C., Lummer M., Navarro L. (2013). Emerging role for RNA based regulation in plant immunity. New Phytol..

[35] Leipe D.D., Koonin E.V., Aravind L. (2004). STAND, a class of P-loop NTPases including animal and plant regulators of programmed cell death: Multiple, complex domain architectures, unusual phyletic patterns, and evolution by horizontal gene transfer. J. Mol. Biol..

[36] Takken F.L.W., Albrecht M., Tameling W.I.L. (2006). Resistance proteins: Molecular switches of plant defence. Curr. Opin. Plant Biol..

[37] Elmore J.M., Lin Z.J.D., Coaker G. (2011). Plant NB-LRR signaling: Upstreams and downstreams. Curr. Opin. Plant Biol..

[38] Kobe B., Kajava A.V. (2001). The leucine-rich repeat as a protein recognition motif. Curr. Opin. Struct. Biol..

[39] Qi D., DeYoung B.J., Innes R.W. (2012). Structure-function analysis of the coiled-coil and leucine-rich repeat domains of the RPS5 disease resistance protein. Plant Physiol..

[40] Van Ooijen G., van den Berg H.A., Cornelissen B.J.C., Takken F.L.W. (2007). Structure and function of resistance proteins in solanaceous plants. Annu. Rev. Phytopathol..

[41] Jones J.D.G., Vance R.E., Dangl J.L. (2016). Intracellular innate immune surveillance devices in plants and animals. Science.

[42] Singh S., Chand S., Singh N.K., Sharma T.R. (2015). Genome-wide distribution, organisation and functional characterization of disease resistance and defence response genes across rice species. PLoS ONE.

[43] Flor H.H. (1971). Current status of the gene-for-gene concept. Annu. Rev. Phytopathol..

[44] Bernoux M., Ve T., Williams S., Warren C., Hatters D., Valkov E., Zhang X., Ellis J.G., Kobe B., Dodds P.N. (2011). Structural and functional analysis of a plant resistance protein TIR domain reveals interfaces for self-association, signaling, and autoregulation. Cell Host Microbe.

[45] Ray S., Singh P.K., Gupta D.K., Mahato A.K., Sarkar C., Rathour R., Singh N.K., Sharma T.R. (2016). Analysis of Magnaporthe oryzae genome reveals a fungal effector, which is able to induce resistance response in transgenic rice line containing resistance gene, *Pi54*. Front. Plant Sci..

[46] Van Der Biezen E.A., Jones J.D.G. (1998). Plant disease-resistance proteins and the gene-for-gene concept. Trends Biochem. Sci..

[47] Van der Hoorn R.A.L., Kamoun S. (2008). From guard to decoy: A new model for perception of plant pathogen effectors. Plant Cell.

[48] Cesari S., Bernoux M., Moncuquet P., Kroj T., Dodds P.N. (2014). A novel conserved mechanism for plant NLR protein pairs: The “integrated decoy” hypothesis. Front. Plant Sci..

[49] Nishimura M.T., Monteiro F., Dangl J.L. (2015). Treasure your exceptions: Unusual domains in immune receptors reveal host virulence targets. Cell.

[50] Liu Y., Wu H., Chen H., Liu Y., He J., Kang H., Sun Z., Pan G., Wang Q., Hu J. (2015). A gene cluster encoding lectin receptor kinases confers broad-spectrum and durable insect resistance in rice. Nat. Biotechnol..

[51] Germain H., Seguin A. (2011). Innate immunity: Has poplar made its BED?. New Phytol..

[52] Das B., Sengupta S., Prasad M., Ghose T.K. (2014). Genetic diversity of the conserved motifs of six bacterial leaf blight resistance genes in a set of rice landraces. BMC Genet..

[53] Kanzaki H., Yoshida K., Saitoh H., Fujisaki K., Hirabuchi A., Alaux L., Fournier E., Tharreau D., Terauchi R. (2012). Arms race co-evolution of *Magnaporthe oryzae AvrPik* and rice *Pik* genes driven by their physical interactions. Plant J..

[54] Cesari S., Thilliez G., Ribot C., Chalvon V., Michel C., Jauneau A., Rivas S., Alaux L., Kanzaki H., Okuyama Y. (2013). The rice resistance protein pair RGA4/RGA5 recognizes the *Magnaporthe oryzae* effectors *Avr-Pia* and *Avr1-CO39* by direct binding. Plant Cell.

[55] Kroj T., Chanclud E., Michel Romiti C., Grand X., Morel J. (2016). Integration of decoy domains derived from protein targets of pathogen effectors into plant immune receptors is widespread. New Phytol..

[56] Bi D., Johnson K.C.M., Zhu Z., Huang Y., Chen F., Zhang Y., Li X. (2011). Mutations in an atypical TIR-NB-LRR-LIM resistance protein confer autoimmunity. Front. Plant Sci..

[57] Costanzo S., Jia Y. (2009). Alternatively spliced transcripts of *Pi-ta* blast resistance gene in *Oryza sativa*. Plant Sci..

[58] Yoshimura S., Yamanouchi U., Katayose Y., Toki S., Wang Z.X., Kono I., Kurata N., Yano M., Iwata N., Sasaki T. (1998). Expression of *Xa1*, a bacterial blight-resistance gene in rice, is induced by bacterial inoculation. Proc. Natl. Acad. Sci. USA.

[59] Gupta S.K., Rai A.K., Kanwar S.S., Sharma T.R. (2012). Comparative analysis of zinc finger proteins involved in plant disease resistance. PLoS ONE.

[60] Sarris P.F., Cevik V., Dagdas G., Jones J.D.G., Krasileva K.V. (2016). Comparative analysis of plant immune receptor architectures uncovers host proteins likely targeted by pathogens. BMC Biol..

[61] Saxena I., Srikanth S., Chen Z. (2016). Cross talk between H_2_O_2_ and interacting signal molecules under plant stress response. Front. Plant Sci..

[62] Sinha A.K., Jaggi M., Raghuram B., Tuteja N. (2011). Mitogen-activated protein kinase signaling in plants under abiotic stress. Plant Signal. Behav..

[63] Reyna N.S., Yang Y. (2006). Molecular analysis of the rice MAP kinase gene family in relation to *Magnaporthe grisea* infection. Mol. Plant Microbe Interact..

[64] Mishra A.K., Sharma K., Mishra R.S. (2012). Elicitor recognition, signal transduction and induced resistance in plants. J. Plant Int..

[65] Tripathy B.C., Oelmuller R. (2012). Reactive oxygen species generation and signaling in plants. Plant Signal. Behav..

[66] Delledonne M., Xia Y., Dixon R.A., Lamb C. (1998). Nitric oxide functions as a signal in plant disease resistance. Nature.

[67] Bari R., Jones J.D. (2009). Role of plant hormones in plant defense responses. Plant Mol. Biol..

[68] Yang D.L., Yang Y., He Z. (2013). Roles of plant hormones and their interplay in rice immunity. Mol. Plant.

[69] Yuan Y., Zhong S., Li Q., Zhu Z., Lou Y., Wang L., Wang J., Wang M., Li Q., Yang D. (2007). Functional analysis of rice *NPR1*-like genes reveals that *OsNPR1/NH1* is the rice orthologue conferring disease resistance with enhanced herbivore susceptibility. Plant Biotechnol. J..

[70] Cai M., Qiu D., Yuan T., Ding X., Li H., Duan L., Xu C., Li X., Wang S. (2008). Identification of novel pathogen-responsive cis-elements and their binding proteins in the promoter of *OsWRKY13*, a gene regulating rice disease resistance. Plant Cell Environ..

[71] Matsushita A., Inoue H., Goto S., Nakayama A., Sugano S., Hayashi N., Takatsuji H. (2013). The nuclear ubiquitin proteasome degradation affects *WRKY45* function in the rice defense program. Plant J..

[72] Robert-Seilaniantz A., Grant M., Jones J.D. (2011). Hormone crosstalk in plant disease and defense: More than just jasmonate-salicylate antagonism. Annu. Rev. Phytopathol..

[73] Liu W., Wang G.L. (2016). Plant innate immunity in rice: A defense against pathogen infection. Natl. Sci. Rev..

[74] Taniguchi S., Hosokawa-Shinonaga Y., Tamaoki D., Yamada S., Akimitsu K., Gomi K. (2014). Jasmonate induction of the monoterpene linalool confers resistance to rice bacterial blight and its biosynthesis is regulated by JAZ protein in rice. Plant Cell Environ..

[75] De Vleesschauwer D., Xu J., Hofte M. (2014). Making sense of hormone-mediated defense networking: From rice to *Arabidopsis*. Front. Plant Sci..

[76] De Vleesschauwer D., Gheysen G., Hofte M. (2013). Hormone defense networking in rice: Tales from a different world. Trends Plant Sci..

[77] De Vleesschauwer D., Seifi H.S., Filipe O., Haeck A., Huu S.N., Demeestere K., Hofte M. (2016). The DELLA protein SLR1 integrates and amplifies salicylic acid and jasmonic acid dependent innate immunity in rice. Plant Physiol..

[78] Jiang C.J., Shimono M., Sugano S., Kojima M., Liu X., Inoue H., Sakakibara H., Takatsuji H. (2013). Cytokinins act synergistically with salicylic acid to activate defense gene expression in rice. Mol. Plant Microbe Interact..

[79] Fu J., Liu H., Li Y., Yu H., Li X., Xiao J., Wang S. (2011). Manipulating broad-spectrum disease resistance by suppressing pathogen-induced auxin accumulation in rice. Plant Physiol..

[80] Zhang J., Peng Y., Guo Z. (2008). Constitutive expression of pathogen-inducible *OsWRKY31* enhances disease resistance and affects root growth and auxin response in transgenic rice plants. Cell Res..

[81] Belkhadir Y., Jaillais Y., Epple P., Balsemao-Pires E., Dangl J.L., Chory J. (2012). Brassinosteroids modulate the efficiency of plant immune responses to microbe-associated molecular patterns. Proc. Natl. Acad. Sci. USA.

[82] Asselbergh B., De Vleesschauwer D., Hofte M. (2008). Global switches and fine-tuning-ABA modulates plant pathogen defense. Mol. Plant Microbe Interact..

[83] Cao F.Y., Yoshioka K., Desveaux D. (2011). The roles of ABA in plant-pathogen interactions. J. Plant Res..

[84] Rawat N. (2016). Understanding disease resistance signaling in rice against various pests and pathogens. Austin J. Plant Biol..

[85] Iwai T., Seo S., Mitsuhara I., Ohashi Y. (2007). Probenazole-induced accumulation of salicylic acid confers resistance to *Magnaporthe grisea* in adult rice plants. Plant Cell Physiol..

[86] Mou Z., Fan W., Dong X. (2003). Inducers of plant systemic acquired resistance Regulate *NPR1* function through redox changes. Cell.

[87] Tada Y., Spoel S.H., Pajerowska-Mukhtar K., Mou Z., Song J., Wang C., Zuo J., Dong X. (2008). Plant immunity requires conformational charges of *NPR1* via S-nitrosylation and thioredoxins. Science.

[88] Johnson C., Boden E., Arias J. (2003). Salicylic acid and *NPR1* induce the recruitment of trans-activating TGA factors to a defense gene promoter in *Arabidopsis*. Plant Cell.

[89] Wang G., Ding X., Yuan M., Qiu D., Li X., Xu C., Wang S. (2006). Dual function of rice *OsDR8* gene in disease resistance and thiamine accumulation. Plant Mol. Biol..

[90] Sugano S., Jiang C.J., Miyazawa S.I., Masumoto C., Yazawa K., Hayashi N., Shimono M., Nakayama A., Miyao M., Takatsuji H. (2010). Role of *OsNPR1* in rice defense program as revealed by genome-wide expression analysis. Plant Mol. Biol..

[91] Chern M., Fitzgerald H.A., Canlas P.E., Navarre D.A., Ronald P.C. (2005). Overexpression of a rice *NPR1* homolog leads to constitutive activation of defense response and hypersensitivity to light. Mol. Plant Microbe Interact..

[92] Shimono M., Sugano S., Nakayama A., Jiang C.J., Ono K., Toki S., Takatsuji H. (2007). Rice *WRKY45* plays a crucial role in Benzothiadiazole-inducible blast resistance. Plant Cell.

[93] Inoue H., Hayashi N., Matsushita A., Xinqiong L., Nakayama A., Sugano S., Jiang C.J., Takatsuji H. (2013). Blast resistance of CC-NB-LRR protein *Pb1* is mediated by *WRKY45* through protein-protein interaction. Proc. Natl. Acad. Sci. USA.

[94] Tao Z., Liu H., Qiu D., Zhou Y., Li X., Xu C., Wang S. (2009). A pair of allelic WRKY genes plays opposite roles in rice-bacteria interactions. Plant Physiol..

[95] Cheng H., Liu H., Deng Y., Xiao J., Li X., Wang S. (2015). The *WRKY45-2WRKY13WRKY42* transcriptional regulatory cascade is required for rice resistance to fungal pathogen. Plant Physiol..

[96] Qiu D., Xiao J., Ding X., Xiong M., Cai M., Cao Y., Li X., Xu C., Wang S. (2007). *OsWRKY13* mediates rice disease resistance by regulating defense-related genes in salicylate- and jasmonate-dependent signaling. Mol. Plant Microbe Interact..

[97] Jimmy J.L., Babu S. (2015). Role of OsWRKY transcription factors in rice disease resistance. Trop. Plant Pathol..

[98] Chujo T., Takai R., Akimoto-Tomiyama C., Ando S., Minami E., Nagamura Y., Kaku H., Shibuya N., Yasuda M., Nakashita H. (2007). Involvement of the elicitor-induced gene *OsWRKY53* in the expression of defense-related genes in rice. Biochim. Biophys. Acta.

[99] Chujo T., Miyamoto K., Ogawa S., Masuda Y., Shimizu T., Kishi-Kaboshi M., Takahashi A., Nishizawa Y., Minami E., Nojiri H. (2014). Overexpression of phosphomimic mutated *OsWRKY53* leads to enhanced blast resistance in rice. PLoS ONE.

[100] Wang H., Hao J., Chen X., Hao Z., Wang X., Lou Y., Peng Y., Guo Z. (2007). Overexpression of rice *WRKY89* enhances ultraviolet B tolerance and disease resistance in rice plants. Plant Mol. Biol..

[101] Abbruscato P., Nepusz T., Mizzi L., Del Corvo M., Morandini P., Fumasoni I., Michel C., Paccanaro A., Guiderdoni E., Schaffrath U. (2012). *OsWRKY22*, a monocot wrky gene, plays a role in the resistance response to blast. Mol. Plant Pathol..

[102] Chujo T., Miyamoto K., Shimogawa T., Shimizu T., Otake Y., Yokotani N., Nishizawa Y., Shibuya N., Nojiri H., Yamane H. (2013). *OsWRKY28*, a PAMP-responsive transrepressor, negatively regulates innate immune responses in rice against rice blast fungus. Plant Mol. Biol..

[103] Liu X., Bai X., Wang X., Chu C. (2007). *OsWRKY71*, a rice transcription factor, is involved in rice defense response. J. Plant Physiol..

[104] Peng X., Wang H., Jang J.C., Xiao T., He H., Jiang D., Tang X. (2016). *OsWRKY80*-*OsWRKY4* module as a positive regulatory circuit in rice resistance against *Rhizoctonia solani*. Rice.

[105] Peng X., Hu Y., Tang X., Zhou P., Deng X., Wang H., Guo Z. (2012). Constitutive expression of rice *WRKY30* gene increases the endogenous jasmonic acid accumulation, PR gene expression and resistance to fungal pathogens in rice. Planta.

[106] Sun L., Zhang H., Li D., Huang L., Hong Y., Ding X.S., Nelson R.S., Zhou X., Song F. (2013). Functions of rice NAC transcriptional factors, *ONAC122* and *ONAC131*, in defense responses against *Magnaporthe grisea*. Plant Mol. Biol..

[107] Nakashima K., Tran L.S.P., Van Nguyen D., Fujita M., Maruyama K., Todaka D., Ito Y., Hayashi N., Shinozaki K., Yamaguchi-Shinozaki K. (2007). Functional analysis of a NAC-type transcription factor *OsNAC6* involved in abiotic and biotic stress-responsive gene expression in rice. Plant J..

[108] Lin R., Zhao W., Meng X., Wang M., Peng Y. (2007). Rice gene *OsNAC19* encodes a novel NAC-domain transcription factor and responds to infection by *Magnaporthe grisea*. Plant Sci..

[109] Yokotani N., Tsuchida-Mayama T., Ichikawa H., Mitsuda N., Ohme-Takagi M., Kaku H., Minami E., Nishizawa Y. (2014). *OsNAC111*, a blast disease-responsive transcription factor in rice, positively regulates the expression of defense-related genes. Mol. Plant Microbe Interact..

[110] Meng X., Zhao W., Lin R., Wang M., Peng Y.-L. (2005). Identification of a novel rice bZIP-type transcription factor gene, *OsbZIP1*, involved in response to infection of *Magnaporthe grisea*. Plant Mol. Biol. Rep..

[111] Li W., Zhong S., Li G., Li Q., Mao B., Deng Y., Zhang H., Zeng L., Song F., He Z. (2011). Rice RING protein *OsBBI1* with E3 ligase activity confers broad-spectrum resistance against *Magnaporthe oryzae* by modifying the cell wall defence. Cell Res..

[112] Peng H., Zhang Q., Li Y., Lei C., Zhai Y., Sun X., Sun D., Sun Y., Lu T. (2009). A putative leucine-rich repeat receptor kinase, *OsBRR1*, is involved in rice blast resistance. Planta.

[113] Li W., Zhu Z., Chern M., Yin J., Yang C., Ran L., Cheng M., He M., Wang K., Wang J. (2017). A natural allele of a transcription factor in rice confers broad-spectrum blast resistance. Cell.

[114] Jisha V., Dampanaboina L., Vadassery J., Mithofer A., Kappara S., Ramanan R. (2015). Overexpression of an AP2/ERF type transcription factor *OsEREBP1* confers biotic and abiotic stress tolerance in rice. PLoS ONE.

[115] Cao Y., Song F., Goodman R.M., Zheng Z. (2006). Molecular characterization of four rice genes encoding ethylene-responsive transcriptional factors and their expressions in response to biotic and abiotic stress. J. Plant Physiol..

[116] Xiao W., Liu H., Li Y., Li X., Xu C., Long M., Wang S. (2009). A rice gene of de novo origin negatively regulates pathogen-induced defense response. PLoS ONE.

[117] Cheung M.Y., Zeng N.Y., Tong S.W., Li W.Y.F., Xue Y., Zhao K.J., Wang C., Zhang Q., Fu Y., Sun Z. (2008). Constitutive expression of a rice GTPase-activating protein induces defense responses. New Phytol..

[118] Ono E., Wong H.L., Kawasaki T., Hasegawa M., Kodama O., Shimamoto K. (2001). Essential role of the small GTPase Rac in disease resistance of rice. Proc. Natl. Sci. USA.

[119] Mei C., Qi M., Sheng G., Yang Y. (2006). Inducible Overexpression of a rice allene oxide synthase gene increases the endogenous jasmonic acid level, PR gene expression, and host resistance to fungal infection. Mol. Plant Microbe Interact..

[120] Schaffrath U., Mauch F., Freydl E., Schweizer P., Dudler R. (2000). Constitutive expression of the defense-related *Rir1b* gene in transgenic rice plants confers enhanced resistance to the rice blast fungus *Magnaporthe grisea*. Plant Mol. Biol..

[121] Yamaguchi T., Kuroda M., Yamakawa H., Ashizawa T., Hirayae K., Kurimoto L., Shinya T., Shibuya N. (2009). Suppression of a phospholipase D gene, *OsPLDβ1*, activates defense responses and increases disease resistance in rice. Plant Physiol..

[122] Sawada K., Hasegawa M., Tokuda L., Kameyama J., Kodama O., Kohchi T., Yoshida K., Shinmyo A. (2004). Enhanced resistance to blast fungus and bacterial blight in transgenic rice constitutively expressing *OsSBP*, a rice homologue of mammalian selenium-binding proteins. Biosci. Biotechnol. Biochem..

[123] Kawano Y., Akamatsu A., Hayashi K., Housen Y., Okuda J., Yao A., Nakashima A., Takahashi H., Yoshida H., Wong H.L. (2010). Activation of a Rac GTPase by the NLR family disease resistance protein Pit plays a critical role in rice innate immunity. Cell Host Microbe.

[124] Domingo C., Andres F., Tharreau D., Iglesias D.J., Talon M. (2009). Constitutive Expression of *OsGH3.1* Reduces auxin content and enhances defense response and resistance to a fungal pathogen in rice. Mol. Plant Microbe Interact..

[125] Khush G.S. (1997). Origin, dispersal, cultivation and variation of rice. Plant Mol. Biol..

[126] Brar D.S., Dalmacio R., Elloran R., Aggarwal R., Angeles R., Khush G.S., Khush G.S. (1996). Gene transfer and molecular characterization of introgression from wild Oryza species into rice. Rice Genetics III.

[127] Jena K.K., Khush G.S. (1990). Introgression of genes from *Oryza officinalis* Well ex Watt to cultivated rice, *O. sativa* L.. Theor. Appl. Genet..

[128] Multani D.S., Jena K.K., Brar D.S., Delos Reyes B.G., Angeles E.R., Khush G.S. (1994). Development of monosomic alien addition lines and introgression of genes from *Oryza australiensis* Domin to cultivated rice, *O. sativa* L.. Theor. Appl. Genet..

[129] Wang G.L., Mackill D.J., Bonman J.M., McCouch S.R., Champoux M.C., Nelson R.J. (1994). RFLP mapping of genes conferring complete and partial resistance to blast in a durably resistant rice cultivar. Genetics.

[130] Young N.D. (1996). QTL mapping and quantitative disease resistance in plants. Annu. Rev. Phytopathol..

[131] Wolfe M.S., Jacobs T., Parleviet J.E. (1993). Can the strategic use of disease resistant hosts protect their inherent durability?. Durability of Disease Resistance.

[132] Simmonds N.W. (1993). Introgression and incorporation. Strategies for the use of crop genetic resources. Biol. Rev..

[133] Thippeswamy S., Chandramohan Y., Zameema S., Srinivas B., Padmaja D., Pravalika K. (2016). Identification of blast resistant rice (*Oryza sativa* L.) genotypes in indigenous and exotic germplasm and validation of pi gene linked molecular markers. Electron. J. Plant Breed..

[134] Chen S., Lin X.H., Xu C.G., Zhang Q. (2000). Improvement of bacterial blight resistance of *Minghui63’*, an elite restorer line of hybrid rice, by molecular marker-assisted selection. Crop Sci..

[135] Bonman J.M. (1992). Durable resistance to rice blast disease-environmental influences. Euphytica.

[136] Tan G.X., Ren X., Weng Q.M., Shi Z.Y., Zhu L.L., He G.C. (2004). Mapping of a new resistance gene to bacterial blight in rice line introgressed from *Oryza officinalis*. Acta Genet. Sin..

[137] Young N.D., Tanksley S.D. (1989). RFLP analysis of the size of chromosomal segments retained around the Tm-2 locus of tomato during backcross breeding. Theor. Appl. Genet..

[138] Hussain B. (2015). Modernization in plant breeding approaches for improving biotic stress resistance in crop plants. Turk. J. Agric. For..

[139] Steffan P., Borgen A., Lazzaro M., Backes G., Torp A.M., Rasmussen S.K. Marker assisted breeding and mass selection of wheat composite cross populations. Proceedings of the 10 Year’s Anniversary Conference Organic Plant Breeding: What Makes the Difference.

[140] McDonald B.A. (2014). Using dynamic diversity to achieve durable disease resistance in agricultural ecosystems. Trop. Plant Pathol..

[141] Keneni G., Bekele E., Imtiaz M., Dagne K. (2012). Genetic vulnerability of modern crop cultivars: Causes, mechanism and remedies. Int. J. Plant Res..

[142] Mundt C.C. (2014). Durable resistance: A key to sustainable management of pathogens and pests. Infect. Genet. Evol..

[143] Sattari A., Fakheri B., Hassan F.S.C., Noroozi M. (2014). Blast resistance in rice: A review of breeding and biotechnology. Int. J. Agric. Crop Sci..

[144] Ashizawa T., Zenbayashi K., Koizumi S. (2001). Development of a simulation model for forecasting rice blast epidemics in multiline. Jpn. J. Phytopathol..

[145] Ishizaki K., Hoshi T., Abe S., Sasaki Y., Kobayashi K., Kasaneyama H., Matsui T., Azuma S. (2005). Breeding of blast resistant lines in rice variety “Koshihikari” and evaluation of their characters. Breed. Sci..

[146] Zhang J., Li X., Jiang G., Xu Y., He Y.Q. (2006). Pyramiding of *Xa7* and *Xa21* for the improvement of disease resistance to bacterial blight in hybrid rice. Plant Breed..

[147] Petit-Houdenot Y., Fudal I. (2017). Complex interactions between fungal avirulence genes and their corresponding plant resistance genes and consequences for disease resistance management. Front. Plant Sci..

[148] Langridge P., Lagudah E.S., Holton T.A., Appels R., Sharp P.J., Chalmers K.J. (2001). Trends in genetic and genome analyses in wheat: A review. Aust. J. Agric. Res..

[149] Hayashi K., Yoshida H., Ashikawa I. (2006). Development of PCR-based allele-specific and InDel marker sets for nine rice blast resistance genes. Theor. Appl. Genet..

[150] Barman S.R., Gowda M., Venu R.C., Chattoo B.B. (2004). Identification of a major blast resistance gene in the rice cultivar “Tetep”. Plant Breed.

[151] Lin F., Chen S., Que Z., Wang L., Liu X., Pan Q. (2007). The blast resistance gene *Pi37* encodes a nucleotide binding site leucine-rich repeat protein and is a member of a resistance gene cluster on rice chromosome 1. Genetics.

[152] Nguyen T.T.T., Koizumi S., La T.N., Zenbayashi K.S., Ashizawa T., Yasuda N., Imazaki I., Miyasaka A. (2006). *Pi35(t)*, a new gene conferring partial resistance to leaf blast in the rice cultivar Hokkai 188. Theor. Appl. Genet..

[153] Sallaud C., Lorieux M., Roumen E., Tharreau D., Berruyer R., Svestasrani P., Garsmeur O., Ghesquiere A., Notteghem J.L. (2003). Identification of five new blast resistance genes in the highly blast-resistant rice variety IR64 using a QTL mapping strategy. Theor. Appl. Genet..

[154] Zhu M., Wang L., Pan Q. (2004). Identification and characterization of a new blast resistance gene located on rice chromosome 1 through linkage and differential analyses. Phytopathology.

[155] Fukuta Y., Yanoria M.J.T., Mercado-Escueta D., Ebron L.A., Fujita Y., Araki E., Khush G.S. (2004). Quantitative trait loci (QTL) reactions to rice blast isolates from Japan and the Philippines. Rice Blast: Interaction with Rice and Control.

[156] Mir G.N., Khush G.S. (1990). Genetics of resistance to bacterial blight in rice cultivar *DV 86*. Crop Res..

[157] Wu X., Li X., Xu C., Wang S. (2008). Fine genetic mapping of *xa24*, a recessive gene for resistance against *Xanthomonas oryzae* pv.*oryzae* in rice. Theor. Appl. Genet..

[158] Chen X.W., Li S.G., Xu J.C., Zhai W.X., Ling Z.Z., Ma B.T., Wang Y.P., Wang W.M., Cao G., Ma Y.Q. (2004). Identification of two blast resistance genes in a rice variety, Digu. J. Phytopathol..

[159] Lei C., Hao K., Yang Y., Ma J., Wang S., Wang J., Cheng Z., Zhao S., Zhang X., Guo X. (2013). Identification and fine mapping of two blast resistance genes in rice cultivar 93-11. Crop J..

[160] Zhou J.H., Wang J.L., Xu J.C., Lei C.L., Ling Z.Z. (2004). Identification and mapping of a rice blast resistance gene *Pi-g(t)* in the cultivar Guangchangzhan. Plant Pathol..

[161] Tabien R.E., Li Z., Paterson A.H., Marchetti M.A., Stansel J.W., Pinson S.R.M., Park W.D. (2000). Mapping of four major rice blast resistance genes from ‘Lemont’ and ‘Teqing’ and evaluation of their combinatorial effect for field resistance. Theor. Appl. Genet..

[162] Pan Q., Wang L., Ikehashi H., Tanisaka T. (1996). Identification of a new blast resistance gene in the indica rice cultivar Kasalath using Japanese differential cultivars and isozyme markers. Phytopathology.

[163] Pan Q. H., Wang L., Ikehashi H., Yamagata H., Tanisaka T. (1998). Identification of two new genes conferring resistance to rice blast in the Chinese native cultivar “*Maowangu*”. Plant Breed..

[164] Ogawa T., Yamamoto T., Banta S.J. (2008). Inheritance of resistance to bacterial blight in rice. Rice Genetics I: (In 2 Parts), Proceedings of the International Rice Genetics Symposium, Manila, Philippines, 27–31 May 1985.

[165] Goto T., Matsumoto T., Furuya N., Tsuchiya K., Yoshimura A. (2009). Mapping of bacterial blight resistance gene *Xa11* on rice chromosome 3. Jpn. Agric. Res. Quart..

[166] Busungu C., Taura S., Sakagami J.I., Ichitani K. (2016). Identification and linkage analysis of a new rice bacterial blight resistance gene from *XM14*, a mutant line from IR24. Breed. Sci..

[167] Liang Z., Wang L., Pan Q. (2016). A new recessive gene conferring resistance against rice blast. Rice.

[168] Sakaguchi S. (1967). Linkage studies on the resistance to bacterial leaf blight, *Xanthomonas oryzae* (Uyeda et Ishiyama) Dowson, in rice. Bull. Natl. Inst. Agric. Sci. Ser. D.

[169] Yoshimura S., Umehara Y., Kurata N., Nagamura Y., Sasaki T., Minobe Y., Iwata N. (1996). Identification of a YAC clone carrying the *Xa1* allele, a bacterial blight resistance gene in rice. Theor. Appl. Genet..

[170] He Q., Li D., Zhu Y., Tan M., Zhang D., Lin X. (2006). Fine mapping of *Xa2*, a bacterial blight resistance gene in rice. Mol. Breed..

[171] Ogawa T., Morinaka T., Fujii K., Kimura T. (1978). Inheritance of resistance of rice varieties Kogyoku and Java 14 to bacterial group V of *Xanthomonas oryzae*. Jpn. J. Phytopathol..

[172] Taura S., Ogawa T., Tabien R.E., Khush G.S., Yoshimura A., Omura T. (1987). The specific reaction of Taichung Native 1 to Philippine races of bacterial blight and inheritance of resistance to race 5 (PXO 112). Rice Genet. Newsl..

[173] Tan Z., Zhang Q., Zhu L., Wang C. (1998). RFLP Mapping of a Rice bacterial blight resistance gene X_ (# alpha#-14). Hereditas.

[174] Wang C., Wen G., Lin X., Liu X., Zhang D. (2009). Identification and fine mapping of the new bacterial blight resistance gene, *Xa31(t)* in rice. Eur. J. Plant Pathol..

[175] Terashima T., Fukuoka S., Saka N., Kudo S. (2008). Mapping of a blast field resistance gene *Pi39(t)* of elite rice strain Chubu 111. Plant Breed..

[176] Fukuoka S., Okuno K., Kawase M. (2007). Rice Blast Disease Gene Pi21, Resistance Gene pi21 and Utilization Thereof. U.S. Patent.

[177] Shinoda H., Toriyama K., Yunoki T., Ezuka A., Sakurai Y. (1971). Studies on the varietal resistance of rice to blast 6. Linkage relationship of blast resistance genes. Jpn. Chugoku Nogyo Shikengo Fukuyama Bull. Ser. A.

[178] Causse M.A., Fulton T.M., Cho Y.G., Ahn S.N., Chunwongse J., Wu K., Xiao J., Yu Z., Ronald P.C., Harrington S.E. (1994). Saturated molecular map of the rice genome based on an interspecific backcross population. Genetics.

[179] Iyer A.S., McCouch S.R. (2004). The rice bacterial blight resistance gene *xa5* encodes a novel form of disease resistance. Mol. Plant Microbe Interact..

[180] Blair M.W., Garris A.J., Iyer A.S., Chapman B., Kresovich S., McCouch S.R. (2003). High resolution genetic mapping and candidate gene identification at the *xa5* locus for bacterial blight resistance in rice (*Oryza sativa* L.). Theor. Appl. Genet..

[181] Ahn S.N., Kim Y.K., Han S.S., Choi H.C., Moon H.P., McCouch S.R. (1996). Molecular mapping of a gene for resistance to a Korean isolate of rice blast. Rice Genet Newsl..

[182] Naqvi N.I., Chattoo B.B. (1996). Development of a sequence characterized amplified region (SCAR) based indirect selection method for a dominant blast-resistance gene in rice. Genome.

[183] Sidhu G.S., Khush G.S., Mew T.W. (1978). Genetic analysis of bacterial blight resistance in seventy-four cultivars of rice, *Oryza sativa* L.. Theor. Appl. Genet..

[184] Kaji R., Ogawa T. (1995). Identification of the located chromosome of the resistance gene, *Xa7*, to bacterial leaf blight in rice. Breed. Sci..

[185] Porter B.W., Chittoor J.M., Yano M., Sasaki T., White F.F. (2003). Development and mapping of markers linked to the rice bacterial blight resistance gene. Crop Sci..

[186] Chen S., Huang Z., Zeng L., Yang J., Liu Q., Zhu X. (2008). High-resolution mapping and gene prediction of *Xanthomonas oryzae* pv.*oryzae* resistance gene *Xa7*. Mol. Breed..

[187] Gu K., Tian D., Yang F., Wu L., Sreekala C., Wang D., Wang G.-L., Yin Z. (2004). High-resolution genetic mapping of *Xa27(t)*, a new bacterial blight resistance gene in rice, *Oryza sativa* L.. Theor. Appl. Genet..

[188] Korinsak S., Sriprakhon S., Sirithanya P., Jairin J., Vanavichit A., Toojinda T. (2009). Identification of microsatellite markers (SSR) linked to a new bacterial blight resistance gene *xa33(t)* in rice cultivar ‘Ba7’. Maejo Int. J. Sci. Technol..

[189] Deng Y., Zhu X., Shen Y., He Z. (2006). Genetic characterization and fine mapping of the blast resistance locus *Pigm(t)* tightly linked to *Pi2* and *Pi9* in a broad-spectrum resistant Chinese variety. Theor. Appl. Genet..

[190] Zhou B., Qu S., Liu G., Dolan M., Sakai H., Lu G., Bellizzi M., Wang G.-L. (2006). The eight amino-acid differences within three leucine-rich repeats between *Pi2* and *Piz-t* resistance proteins determine the resistance specificity to *Magnaporthe grisea*. Mol. Plant Microbe Interact..

[191] Jeung J.U., Kim B.R., Cho Y.C., Han S.S., Moon H.P., Lee Y.T., Jena K.K. (2007). A novel gene, *Pi40(t)*, linked to the DNA markers derived from NBS-LRR motifs confers broad spectrum of blast resistance in rice. Theor. Appl. Genet..

[192] Koide Y., Telebanco-Yanoria M.J., Fukuta Y., Kobayashi N. (2013). Detection of novel blast resistance genes, *Pi58(t)* and *Pi59(t)*, in a Myanmar rice landrace based on a standard differential system. Mol. Breed..

[193] Qu S., Liu G., Zhou B., Bellizzi M., Zeng L., Dai L., Han B., Wang G.-L. (2006). The broad-spectrum blast resistance gene *Pi9* encodes a nucleotide-binding site leucine-rich repeat protein and is a member of a multigene family in rice. Genetics.

[194] Wang Y., Wang D., Deng X., Liu J., Sun P., Liu Y., Huang H., Jiang N., Kang H., Ning Y. (2012). Molecular mapping of the blast resistance genes *Pi2-1* and *Pi51(t)* in the durably resistant rice “Tianjingyeshengdao”. Phytopathology.

[195] Chen X., Shang J., Chen D., Lei C., Zou Y., Zhai W., Liu G., Xu J., Ling Z., Cao G. (2006). AB lectin receptor kinase gene conferring rice blast resistance. Plant J..

[196] Wu J.-L., Fan Y.-Y., Li D.-B., Zheng K.-L., Leung H., Zhuang J.-Y. (2005). Genetic control of rice blast resistance in the durably resistant cultivar Gumei 2 against multiple isolates. Theor. Appl. Genet..

[197] Fjellstrom R., Conaway-Bormans C.A., McClung A.M., Marchetti M.A., Shank A.R., Park W.D. (2004). Development of DNA markers suitable for marker assisted selection of three genes conferring resistance to multiple pathotypes. Crop Sci..

[198] Hayasaka H. (1995). Mapping genes conferring rice blast resistance in rice variety Kasalath using RFLP markers. II. Linkage analysis of the resistance gene on chromosome 6. Breed. Sci..

[199] Ballini E., Morel J., Droc G., Price A., Courtois B., Notteghem J., Tharreau D.A. (2008). Genome-wide meta-analysis of rice blast resistance genes and quantitative trait loci provides new insights into partial and complete resistance. Mol. Plant Microbe Interact..

[200] Jiang N., Li Z., Wu J., Wang Y., Wu L., Wang S., Wang D., Wen T., Liang Y., Sun P. (2012). Molecular mapping of the *Pi2/9* allelic gene *Pi2-2* conferring broad-spectrum resistance to *Magnaporthe oryzae* in the rice cultivar Jefferson. Rice.

[201] Zhu X., Chen S., Yang J., Zhou S., Zeng L., Han J., Su J., Wang L., Pan Q. (2012). The identification of *Pi50(t)*, a new member of the rice blast resistance *Pi2*/*Pi9* multigene family. Theor. Appl. Genet..

[202] Vikal Y., Chawla H., Sharma R., Lore J.S., Singh K. (2014). Mapping of bacterial blight resistance gene *xa8* in rice (*Oryza sativa* L.). Indian J. Genet. Plant Breed..

[203] Pan Q.H., Tanisaka T., Ikehashi H. (1995). Studies on the genetics and breeding of blast resistance in rice IV. Gene analysis for the blast resistance of a indica variety Kasalath. Breed. Sci..

[204] Chu Z., Fu B., Yang H., Xu C., Li Z., Sanchez A., Park Y.J., Bennetzen J.L., Zhang Q., Wang S. (2006). Targeting *xa13*, a recessive gene for bacterial blight resistance in rice. Theor. Appl. Genet..

[205] Ogawa T., Lin L., Tabien R.E., Khush G.S. (1987). A new recessive gene for resistance to bacterial blight of rice. Rice Genet. Newsl..

[206] Sanchez A.C., Ilag L.L., Yang D., Brar D.S., Ausubel F., Khush G.S., Yano M., Sasaki T., Li Z., Huang N. (1999). Genetic and physical mapping of *xa13*, a recessive bacterial blight resistance gene in rice. Theor. Appl. Genet..

[207] Zhang G., Angeles E.R., Abenes M.L.P., Khush G.S., Huang N. (1996). RAPD and RFLP mapping of the bacterial blight resistance gene *xa*-*13* in rice. Theor. Appl. Genet..

[208] Yang B., Sugio A., White F.F. (2006). *Os8N3* is a host disease-susceptibility gene for bacterial blight of rice. Proc. Natl. Acad. Sci. USA.

[209] Berruyer R., Adreit H., Milazzo J., Gaillard S., Berger A., Dioh W., Lebrun M.-H., Tharreau D. (2003). Identification and fine mapping of *Pi33*, the rice resistance gene corresponding to the *Magnaporthe grisea* avirulence gene *ACE1*. Theor. Appl. Genet..

[210] Zhu L.-H. (1993). Construction of a molecular map of rice and gene mapping using a double-haploid population of a cross between indica and japonica varieties. Rice Genet. Newsl..

[211] Liu X.Q., Wang L., Chen S., Lin F., Pan Q.H. (2005). Genetic and physical mapping of *Pi36(t)*, a novel rice blast resistance gene located on rice chromosome 8. Mol. Genet. Genom..

[212] He X., Liu X., Wang L., Wang L., Lin F., Cheng Y., Chen Z., Liao Y., Pan Q. (2012). Identification of the novel recessive gene *pi55(t)* conferring resistance to *Magnaporthe oryzae*. Sci. China Life Sci..

[213] Liu B., Zhang S., Zhu X., Yang Q., Wu S., Mei M., Mauleon R., Leach J., Mew T., Leung H. (2004). Candidate defense genes as predictors of quantitative blast resistance in rice. Mol. Plant Microbe Interact..

[214] Pan Q.-H., Hu Z.-D., Takatoshi T., Wang L. (2003). Fine mapping of the blast resistance gene *Pi15*, linked to *Pii*, on rice chromosome 9. Acta Bot. Sin..

[215] Kinoshita T., Kiyosawa S. (1997). Some considerations on linkage relationships between *Pii* and *Piz* in the blast resistance of rice. Rice Genet. Newsl..

[216] Mackill D.J., Bonman J.M. (1992). Inheritance of blast resistance in near-isogenic lines of rice. Phytopathology.

[217] Jeon J.-S., Chen D., Yi G.-H., Wang G.L., Ronald P.C. (2003). Genetic and physical mapping of *Pi5(t)*, a locus associated with broad-spectrum resistance to rice blast. Mol. Genet. Genom..

[218] Liu Y., Liu B., Zhu X., Yang J., Bordeos A., Wang G., Leach J.E., Leung H. (2013). Fine-mapping and molecular marker development for *Pi56(t)*, a NBS-LRR gene conferring broad-spectrum resistance to *Magnaporthe oryzae* in rice. Theor. Appl. Genet..

[219] Xiang Y., Cao Y., Xu C., Li X., Wang S. (2006). *Xa3*, conferring resistance for rice bacterial blight and encoding a receptor kinase-like protein, is the same as *Xa26*. Theor. Appl. Genet..

[220] Ezuka A., Horino O., Toriyama K. (1975). Inheritance of resistance of rice variety Wase Aikoku 3 to *Zanthomonas oryzae*. Bulletin.

[221] Yoshimura S., Yoshimura A., Iwata N., McCouch S.R., Abenes M.L., Baraoidan M.R., Mew T.W., Nelson R.J. (1995). Tagging and combining bacterial blight resistance genes in rice using RAPD and RFLP markers. Mol. Breed..

[222] Sun X., Yang Z., Wang S., Zhang Q. (2003). Identification of a 47-kb DNA fragment containing *Xa4*, a locus for bacterial blight resistance in rice. Theor. Appl. Genet..

[223] Mew T.W., Cruz V., Reyes R.C. (1982). Interaction of *Xanthomonas campestris* pv. *oryzae* and a resistant rice cultivar. Phytopathology.

[224] Yoshimura A., Mew T.W., Khush G.S., Omura T. (1983). Inheritance of resistance to bacterial blight in rice cultivar Cas 209. Phytopathology.

[225] Gu K., Sangha J.S., Li Y., Yin Z. (2008). High-resolution genetic mapping of bacterial blight resistance gene *Xa10*. Theor. Appl. Genet..

[226] Song W.-Y., Wang G.-L., Chen L.-L., Kim H.-S., Pi L.-Y., Holsten T., Gardner J., Wang B., Zhai W.-X., Zhu L.-H. (1995). A receptor kinase-like protein encoded by the rice disease resistance gene, *Xa21*. Science.

[227] Khush G.S., Bacalangco E., Ogawa T. (1991). A new gene for resistance to bacterial blight from *O. longistaminata*. Rice Genet. Newsl..

[228] Ronald P.C., Albano B., Tabien R., Abenes L., Wu K., McCouch S., Tanksley S.D. (1992). Genetic and physical analysis of the rice bacterial blight disease resistance locus, *Xa21*. Mol. Gen. Genet..

[229] Lin X.H., Zhang D.P., Xie Y.F., Gao H.P., Zhang Q. (1996). Identifying and mapping a new gene for bacterial blight resistance in rice based on RFLP markers. Phytopathology.

[230] Wang C., Tan M., Xu X., Wen G., Zhang D., Lin X. (2003). Localizing the bacterial blight resistance gene, *Xa22(t)*, to a 100-kilobase bacterial artificial chromosome. Phytopathology.

[231] Zhang Q., Lin S.C., Zhao B.Y., Wang C.L., Yang W.C., Zhou Y.L., Li D.Y., Chen C.B., Zhu L.H. (1998). Identification and tagging a new gene for resistance to bacterial blight (*Xanthomonas oryzae pv. oryzae*) from *O. rufipogon*. Rice Genet. Newsl..

[232] Jin X.W., Wang C.L., Yang Q., Jiang Q.X., Fan Y.L., Liu G.C., Zhao K.J. (2007). Breeding of near-isogenic line CBB30 and molecular mapping of *Xa30(t)*, a new resistance gene to bacterial blight in rice. Sci. Agric. Sin..

[233] Zheng C.-K., Chun-Lian W., Yuan-Jie Y.U., Liang Y.-T., Kai-Jun Z. (2009). Identification and molecular mapping of *Xa32(t)*, a novel resistance gene for bacterial blight (*Xanthomonas oryzae pv. oryzae*) in rice. Acta Agron. Sin..

[234] Guo S., Zhang D., Lin X. (2010). Identification and mapping of a novel bacterial blight resistance gene *Xa35(t)* originated from *Oryza minuta*. Sci. Agric. Sin..

[235] Zhang F., Zhuo D., Huang L., Wang W., Xu J., Vera Cruz C., Li Z., Zhou Y. (2015). *Xa39*, a novel dominant gene conferring broad-spectrum resistance to *Xanthomonas oryzae* pv*. oryzae* in rice. Plant Pathol..

[236] Kim S.-M., Suh J.-P., Qin Y., Noh T.-H., Reinke R.F., Jena K.K. (2015). Identification and fine-mapping of a new resistance gene, *Xa40*, conferring resistance to bacterial blight races in rice (*Oryza sativa* L.). Theor. Appl. Genet..

[237] Wu Y., Bao Y., Xie L., Su Y., Chu R., He W., Huang J., Wang J., Zhang H. (2013). Fine mapping and identification of blast resistance gene *Pi-hk1* in a broad-spectrum resistant japonica rice landrace. Phytopathology.

[238] Sharma T.R., Madhav M.S., Singh B.K., Shanker P., Jana T.K., Dalal V., Pandit A., Singh A., Gaikwad K., Upreti H.C. (2005). High-resolution mapping, cloning and molecular characterization of the *Pi-kh* gene of rice, which confers resistance to *Magnaporthe grisea*. Mol. Genet. Genomics.

[239] Fuentes J.L., Correa-Victoria F.J., Escobar F., Prado G., Aricapa G., Duque M.C., Tohme J. (2008). Identification of microsatellite markers linked to the blast resistance gene *Pi-1(t)* in rice. Euphytica.

[240] Fujii K., Hayano-Saito Y., Saito K., Sugiura N., Hayashi N., Tsuji T., Izawa T., Iwasaki M. (2000). Identification of a RFLP marker tightly linked to the panicle blast resistance gene, *Pb1*, in rice. Breed. Sci..

[241] Goto I. (1970). Genetic studies on the resistance of rice plant to the blast fungus. I. Inheritance of resistance in crosses Sensho X H-79 and Imochi-shirazu X H-79. Ann. Phytopathol. Soc. Jpn..

[242] Goto I., Jaw Y.-L., Baluch A.A. (1981). Genetic studies on resistance of rice plant to blast fungus. Jpn. J. Phytopathol..

[243] Gowda M., Roy-Barman S., Chattoo B.B. (2006). Molecular mapping of a novel blast resistance gene *Pi38* in rice using SSLP and AFLP markers. Plant Breed..

[244] Yunoki T., Ezuka A., Morinaka T., Sakurai Y., Shinoda H., Toriyama K. (1970). Studies on the varietal resistance to Rice blast. 4. Variation of field resistance due to fungus strains. Bull. Chugoku Agric. Exp. Stn. Ser. A.

[245] Zenbayashi-Sawata K., Ashizawa T., Koizumi S. (2005). *Pi34*-*AvrPi34*: A new gene-for-gene interaction for partial resistance in rice to blast caused by *Magnaporthe grisea*. J. Gen. Plant Pathol..

[246] Kwon S.-W., Cho Y.-C., Kim Y.-G., Suh J.-P., Jeung J.-U., Roh J.-H., Lee S.-K., Jeon J.-S., Yang S.-J., Lee Y.-T. (2008). Development of near-isogenic japonica rice lines with enhanced resistance to *Magnaporthe grisea*. Mol. Cells.

[247] Chauhan R., Farman M., Zhang H.-B., Leong S. (2002). Genetic and physical mapping of a rice blast resistance locus, *Pi-CO39(t)*, that corresponds to the avirulence gene *Avr1-CO39* of *Magnaporthe grisea*. Mol. Genet. Genom..

[248] Chen D.-H., Dela Vina M., Inukai T., Mackill D.J., Ronald P.C., Nelson R.J. (1999). Molecular mapping of the blast resistance gene, *Pi44(t)*, in a line derived from a durably resistant rice cultivar. Theor. Appl. Genet..

[249] Sun P., Liu J., Wang Y., Jiang N., Wang S., Dai Y., Gao J., Li Z., Pan S., Wang D. (2013). Molecular mapping of the blast resistance gene *Pi49* in the durably resistant rice cultivar Mowanggu. Euphytica.

[250] Kaji R., Ogawa T. (1996). RFLP Mapping of Blast Resistance Gene Pi-km in Rice.

[251] Ahn S.-N., Kim Y.-K., Hong H.-C., Han S.-S., Kwon S.-J., Choi H.-C., Moon H.-P., McCouch S.R. (2000). Molecular mapping of a new gene for resistance to rice blast (*Pyricularia grisea* Sacc.). Euphytica.

[252] Huang H., Huang L., Feng G., Wang S., Wang Y., Liu J., Jiang N., Yan W., Xu L., Sun P. (2011). Molecular mapping of the new blast resistance genes *Pi47* and *Pi48* in the durably resistant local rice cultivar Xiangzi 3150. Phytopathology.

[253] Prasad M.S., Kanthi B.A., Balachandran S.M., Seshumadhav M., Mohan K.M., Viraktamath B.C. (2009). Molecular mapping of rice blast resistance gene *Pi-1(t)* in the elite indica variety Samba mahsuri. World J. Microbiol. Biotechnol..

[254] Hua L., Wu J., Chen C., Wu W., He X., Lin F., Wang L., Ashikawa I., Matsumoto T., Wang L. (2012). The isolation of *Pi1*, an allele at the *Pik* locus which confers broad spectrum resistance to rice blast. Theor. Appl. Genet..

[255] Lee K.S., Rasabandith S., Angeles E.R., Khush G.S. (2003). Inheritance of resistance to bacterial blight in 21 cultivars of rice. Phytopathology.

[256] Zhuang J.-Y., Ma W.-B., Wu J.-L., Chai R.-Y., Lu J., Fan Y.-Y., Jin M.-Z., Leung H., Zheng K.-L. (2002). Mapping of leaf and neck blast resistance genes with resistance gene analog, RAPD and RFLP in rice. Euphytica.

[257] Kumar P., Pathania S., Katoch P., Sharma T.R., Plaha P., Rathour R. (2010). Genetic and physical mapping of blast resistance gene *Pi42(t)* on the short arm of rice chromosome 12. Mol. Breed..

[258] Wu K.S., Martinez C., Lentini Z., Tohme J., Chumley F.G., Scolnik P.A., Valent B. (2008). Cloning a blast resistance gene by chromosome walking. Rice Genetics III: (In 2 Parts), Proceedings of the Third International Rice Genetics Symposium, Manila, Philippines, 16–20 October 1995.

[259] Yu Z.H., Mackill D.J., Bonman J.M., Tanksley S.D. (1991). Tagging genes for blast resistance in rice via linkage to RFLP markers. Theor. Appl. Genet..

[260] Inukai T., Nelson R. (1994). Mapping for blast resistance gene H-3 derived from rice cuhivar Pai-Kan-Tao. Rep. Hokkaido Br. CropSol. See Jap. Soc. Breed..

[261] Hayashi K., Yoshida H. (2009). Refunctionalization of the ancient rice blast disease resistance gene *Pit* by the recruitment of a retrotransposon as a promoter. Plant J..

[262] Bryan G.T., Wu K.-S., Farrall L., Jia Y., Hershey H.P., McAdams S.A., Faulk K.N., Donaldson G.K., Tarchini R., Valent B. (2000). A single amino acid difference distinguishes resistant and susceptible alleles of the rice blast resistance gene *Pi-ta*. Plant Cell.

[263] Liu X., Lin F., Wang L., Pan Q. (2007). The in silico map-based cloning of *Pi36*, a rice coiled-coil-nucleotide-binding site leucine-rich repeat gene that confers race-specific resistance to the blast fungus. Genetics.

[264] Li L.-Y., Wang L., Jing J.-X., Li Z.-Q., Lin F., Huang L.-F., Pan Q.-H. (2007). The *Pikm* gene, conferring stable resistance to isolates of *Magnaporthe oryzae*, was finely mapped in a crossover-cold region on rice chromosome 11. Mol. Breed..

[265] Talukder Z.I., McDonald A.J.S., Price A.H. (2005). Loci controlling partial resistance to rice blast do not show marked QTL× environment interaction when plant nitrogen status alters disease severity. New Phytol..

[266] Xu J., Wang J., Ling Z., Zhu L. (2004). Analysis of rice blast resistance genes by QTL mapping. Chin. Sci. Bull..

[267] Albar L., Lorieux M., Ahmadi N., Rimbault I., Pinel A., Sy A.A., Fargette D., Ghesquiere A. (1998). Genetic basis and mapping of the resistance to rice yellow mottle virus. QTLs identification and relationship between resistance and plant morphology. Theor. Appl. Genet..

[268] Djedatin G., Ndjiondjop M.N., Sanni A., Lorieux M., Verdier V., Ghesquiere A. (2016). Identification of novel major and minor QTLs associated with *Xanthomonas oryzae* pv.*oryzae* (African strains) resistance in rice (*Oryza sativa* L.). Rice.

[269] Yang C.D., Zeng D.L., Ma L.Y., Ji Z.J., Guo L.B., Li X.M., Qian Q. (2006). Mapping QTLs for bacterial blight resistance in a DH population from japonica/indica cross of rice (*Oryzae sativa*). Chin. J. Rice Sci..

[270] Fiyaz R.A., Yadav A.K., Krishnan S.G., Ellur R.K., Bashyal B.M., Grover N., Bhowmick P.K., Nagarajan M., Vinod K.K., Singh N.K. (2016). Mapping quantitative trait loci responsible for resistance to Bakanae disease in rice. Rice.

[271] Volante A., Tondelli A., Aragona M., Valente M.T., Biselli C., Desiderio F., Bagnaresi P., Matic S., Gullino M.L., Infantino A. (2017). Identification of bakanae disease resistance loci in japonica rice through genome wide association study. Rice.

[272] Tabien R., Li Z., Paterson A., Marchetti M., Stansel J., Pinson S. (2002). Mapping QTLs for field resistance to the rice blast pathogen and evaluating their individual and combined utility in improved varieties. Theor. Appl. Genet..

[273] Tang D., Wu W., Li W., Lu H., Worland A.J. (2000). Mapping of QTLs conferring resistance to bacterial leaf streak in rice. Theor. Appl. Genet..

[274] Loan L.C., Du P.V., Li Z. (2004). Molecular dissection of quantitative resistance of sheath blight in rice (*Oryza sativa* L.). Omonrice.

[275] Sato H., Matsumoto K., Ota C., Yamakawa T., Kihara J., Mizobuchi R. (2015). Confirming a major QTL and finding additional loci responsible for field resistance to brown spot (*Bipolaris oryzae*) in rice. Breed. Sci..

[276] Bagali P.G., Hittalmani S., Shashidhar S.Y., Shashidhar H.E., Tharreau D., Lebrun M.H., Talbot N.J., Notteghem J.L. (2000). Identification of DNA markers linked to partial resistance for blast disease in rice across four locations. Advances in Rice Blast Research. Developments in Plant Pathology.

[277] Li Y.S., Zhang Y.D., Zhu Z., Zhao L., Wang C.L. (2008). QTL analysis for resistance to rice false smut by using recombinant inbred lines in rice. Chin. J. Rice Sci..

[278] Lopez-Gerena J. (2006). Mapping QTL Controlling Durable Resistance to Rice Blast in the Cultivar Oryzica Llanos 5. Ph.D. Thesis.

[279] Chen H., Wang S., Xing Y., Xu C., Hayes P.M., Zhang Q. (2003). Comparative analyses of genomic locations and race specificities of loci for quantitative resistance to *Pyricularia grisea* in rice and barley. Proc. Natl. Acad. Sci. USA.

[280] Pinson S.R.M., Capdevielle F.M., Oard J.H. (2005). Confirming QTLs and finding additional loci conditioning sheath blight resistance in rice using recombinant inbred lines. Crop Sci..

[281] Loan L.C., Du P., Li Z. (2003). Identification of genes conferring resistance to some Philippine and Vietnamese races of blast. Omonrice.

[282] Liu G., Jia Y., Correa-Victoria F.J., Prado G.A., Yeater K.M., Mcclung A., Correll J.C. (2009). Mapping quantitative trait loci responsible for resistance to sheath blight in rice. Phytopathology.

[283] Ding X., Jiang L., Zhang Y., Sun D.-Z., Zhai H.-Q., Wan J.-M. (2005). Detection and analysis of QTL for resistance to stripe disease in rice, using backcross inbred lines. Acta Agron. Sin..

[284] Katara J.L., Sonah H., Deshmukh R.K., Chaurasia R., Kotasthane A.S. (2010). Molecular analysis of QTLs associated with resistance to brown spot in rice (*Oryza sativa* L.). Indian J. Genet..

[285] Sato H., Ando I., Hirabayashi H., Takeuchi Y., Arase S., Kihara J., Kato H., Imbe T., Nemoto H. (2008). QTL analysis of brown spot resistance in rice (*Oryza sativa* L.). Breed. Sci..

[286] Zhou Y.L., Xie X.W., Zhang F., Wang S., Liu X.Z., Zhu L.H., Xu J., Gao Y.M., Li Z. (2014). Detection of quantitative resistance loci associated with resistance to rice false smut (*Ustilaginoidea virens*) using introgression lines. Plant Pathol..

[287] Chen C.-H., Zheng W., Huang X.-M., Zhang D.-P., Lin X.-H. (2006). Major QTL conferring resistance to rice bacterial leaf streak. Agric. Sci. China.

[288] Kunihiro Y., Qian Q., Sato H., Teng S., Zeng D.-L., Fujimoto K., Zhu L.H. (2002). QTL analysis of sheath blight resistance in rice (*Oryza sativa* L.). Acta Genet. Sin..

[289] Zou J.H., Pan X.B., Chen Z.X., Xu J.Y., Lu J.F., Zhai W.X., Zhu L.H. (2000). Mapping quantitative trait loci controlling sheath blight resistance in two rice cultivars (*Oryza sativa* L.). Theor. Appl. Genet..

[290] Xu Q., Yuan X., Yu H., Wang Y., Tang S., Wei X. (2011). Mapping quantitative trait loci for sheath blight resistance in rice using double haploid population. Plant Breed..

[291] Wang B., Jiang L., Chen L., Lu B., Wang Q., Quang T.L., Fan J., Cheng X., Zhai H., Xu D. (2010). Screening of rice resources against rice black-streaked dwarf virus and mapping of resistant QTL. Acta Agron. Sin..

[292] Pan X., Zou J., Chen Z., Lu J., Yu H., Li H., Wang Z., Pan X., Rush M.C., Zhu L. (1999). Tagging major quantitative trait loci for sheath blight resistance in rice variety, Jasmine 85. Chinese Sci. Bull..

[293] Yu Y.C., Teng S., Zeng D.L., Dong G.J., Qian Q., Huang D.N., Zhu L.W. (2003). Analysis of QTLs for resistance to rice bacterial blight. Chin. J. Rice Sci..

[294] Sato H., Takeuchi Y., Hirabayashi H., Nemoto H., Hirayama M., Kato H., Imbe T., Ando I. (2006). Mapping QTLs for field resistance to rice blast in the Japanese upland rice variety Norin 12. Breed. Sci..

[295] Zhou T., Du L., Wang L., Wang Y., Gao C., Lan Y., Sun F., Fan Y., Wang G., Zhou Y. (2015). Genetic analysis and molecular mapping of QTLs for resistance to rice black-streaked dwarf disease in rice. Sci. Rep..

[296] Li Z., Pinson S.R.M., Marchetti M.A., Stansel J.W., Park W.D. (1995). Characterization of quantitative trait loci (QTLs) in cultivated rice contributing to field resistance to sheath blight (*Rhizoctonia solani*). Theor. Appl. Genet..

[297] Wu S.-J., Zhong H., Zhou Y., Zuo H., Zhou L.-H., Zhu J.-Y., Ji C.-Q., Gu S.-L., Gu M.-H., Liang G.-H. (2009). Identification of QTLs for the resistance to rice stripe virus in the indica rice variety Dular. Euphytica.

[298] Wang C., Su C., Zhai H., Wan J. (2005). Identification of QTLs underlying resistance to a virulent strain of *Xanthomonas oryzae* pv.*oryzae* in rice cultivar DV85.F. Crop Res..

[299] Fukuoka S., Okuno K. (2001). QTL analysis and mapping of *pi21*, a recessive gene for field resistance to rice blast in Japanese upland rice. Theor. Appl. Genet..

[300] Miyamoto M., Yano M., Hirasawa H. (2001). Mapping of quantitative trait loci conferring blast field resistance in the Japanese upland rice variety *Kahei*. Breed. Sci..

[301] Han Y.P., Xing Y.Z., Chen Z.X., Gu S.L., Pan X.B., Chen X.L., Zhang Q.F. (2002). Mapping QTLs for horizontal resistance to sheath blight in an elite rice restorer line, Minghui 63. Acta Genet. Sin..

[302] Sun D.-Z., Jiang L., Zhang Y.-X., Cheng X.-N., Zhai H.-Q., Wan J.-M. (2007). Quantitative trait loci for resistance to stripe disease in rice (*Oryza sativa*). Rice Sci..

[303] Liu Y., Zhu X.Y., Zhang S., Bernardo M., Edwards J., Galbraith D.W., Leach J., Zhang G., Liu B., Leung H. (2011). Dissecting quantitative resistance against blast disease using heterogeneous inbred family lines in rice. Theor. Appl. Genet..

[304] Tan C.-X., Ji X.-M., Yang Y., Pan X.-Y., Zuo S.-M., Zhang Y.-F., Zou J.-H., Chen Z.-X., Zhu L.-H., Pan X.-B. (2005). Identification and marker-assisted selection of two major quantitative genes controlling rice sheath blight resistance in backcross generations. Acta Genet. Sin..

[305] .Xiu-Lan D., Ling J., Shi-Jia L., Chun-Ming W., Liang-Ming C., Zhao-Bang C., Yong-Jian F., Yi-Jun Z., Jian-Min W. (2004). QTL analysis for rice stripe disease resistance gene using recombinant inbred lines (RILs) derived from crossing of *Kinmaze* and *DV85*. Acta Genet. Sin..

[306] Ashkani S., Rafil M.Y., Shabanimofrad M., Miah G., Sahebi M., Azizi P., Tanweer F.A., Akhtar M.S., Nasehi A. (2015). Molecular breeding strategy and challenges towards the improvement of blast disease resistance in rice crop. Front. Plant Sci..

[307] Das A., Soubam D., Singh P.K., Thakur S., Singh N.K., Sharma T.R. (2012). A novel blast resistance gene, *Pi54rh* cloned from wild species of rice, *Oryza rhizomatis* confers broad spectrum resistance to *Magnaporthe oryzae*. Funct. Integr. Genom..

[308] Thakur S., Gupta Y.K., Singh P.K., Rathour R., Variar M., Prashanthi S.K., Singh A.K., Singh U.D., Chand D., Rana J.C. (2013). Molecular diversity in rice blast resistance gene *Pi-ta* makes it highly effective against dynamic population of *Magnaporthe oryzae*. Funct. Integr. Genom..

[309] Thakur S., Singh P.K., Rathour R., Variar M., Prashanthi S.K., Singh A.K., Singh U.D., Chand D., Singh N.K., Sharma T.R. (2013). Positive selection pressure on rice blast resistance allele *Piz-t* makes it divergent in Indian landraces. J. Plant Int..

[310] Thakur S., Singh P.K., Das A., Rathour R., Variar M., Prashanthi S.K., Singh A.K., Singh U.D., Chand D., Singh N.K. (2015). Extensive sequence variation in rice blast resistance gene *Pi54* makes it broad spectrum in nature. Front. Plant Sci..

[311] Devanna N.B., Vijayan J., Sharma T.R. (2014). The blast resistance gene *Pi54of* cloned from Oryza officinalis interacts with Avr-Pi54 through its novel non-LRR domains. PLoS ONE.

[312] Jia Y., Liu G., Costanzo S., Lee S., Dai Y. (2009). Current progress on genetic interactions of rice with rice blast and sheath blight fungi. Front. Agric. China.

[313] Barnwal M.K., Kotasthane A., Magculia N., Mukherjee P.K., Savary S., Sharma A.K., Singh H.B., Singh U.S., Sparks A.H., Variar M. (2013). A review on crop losses, epidemiology and disease management of rice brown spot to identify research priorities and knowledge gaps. Eur. J. Plant Pathol..

[314] Mizobuchi R., Fukuoka S., Tsushima S., Yano M., Sato H. (2016). QTLs for resistance to major rice diseases exacerbated by global warming: Brown spot, bacterial seedling rot and bacterial grain rot. Rice.

[315] Yang C.D., Guo L.B., Li X.M., Ji Z.J., Ma L.Y., Qian Q. (2006). Analysis of QTLs for resistance to rice bakanae disease. Chin. J. Rice Sci..

[316] Hur Y.J., Lee S.B., Kim T.H., Kwon T., Lee J.H., Shin D.J., Park S.K., Hwang U.H., Cho J.H., Yoon Y.N. (2015). Mapping of *qBK1*, a major QTL for bakanae disease resistance in rice. Mol. Breed..

[317] Guo X., Li Y., Fan J., Li L., Huang F., Wang W. (2012). Progress in the study of false smut disease in rice. J. Agric. Sci. Technol. A.

[318] Andargie M., Li J. (2016). *Arabidopsis thaliana:* A model host plant to study plant-pathogen interaction using rice false smut isolates of *Ustilaginoidea virens*. Front. Plant Sci..

[319] Kou Y., Wang S. (2012). Toward an understanding of the molecular basis of quantitative disease resistance in rice. J. Biotechnol..

[320] Nino-Liu D.O., Ronald P.C., Bogdanove A.J. (2006). *Xanthomonas oryzae* pathovars: Model pathogens of a model crop. Mol. Plant Pathol..

[321] Triplett L.R., Cohen S.P., Heffelfinger C., Schmidt C.L., Huerta A.I., Tekete C., Verdier V., Bogdanove A.J., Leach J.E. (2016). A resistance locus in the American heirloom rice variety Carolina Gold Select is triggered by TAL effectors with diverse predicted targets and is effective against African strains of *Xanthomonas oryzae* pv. *oryzicola*. Plant J..

[322] Ham J.H., Melanson R.A., Rush M.C. (2011). Burkholderia glumae: Next major pathogen of rice?. Mol. Plant Pathol..

[323] Hayano-Saito Y., Saito K., Nakamura S., Kawasaki S., Iwasaki M. (2000). Fine physical mapping of the rice stripe resistance gene locus, Stvb-i. Theor. Appl. Genet..

[324] Albar L., Ndjiondjop M.-N., Esshak Z., Berger A., Pinel A., Jones M., Fargette D., Ghesquiere A. (2003). Fine genetic mapping of a gene required for *Rice yellow mottle virus* cell-to-cell movement. Theor. Appl. Genet..

[325] Thiemele D., Boisnard A., Ndjiondjop M.N., Cheron S., Sere Y., Ake S., Ghesquiere A., Albar L. (2010). Identification of a second major resistance gene to rice yellow mottle virus, RYMV2, in the African cultivated rice species, *O. glaberrima*. Theor. Appl. Genet..

[326] Lee J.-H., Muhsin M., Atienza G.A., Kwak D.-Y., Kim S.-M., De Leon T.B., Angeles E.R., Coloquio E., Kondoh H., Satoh K. (2010). Single nucleotide polymorphisms in a gene for translation initiation factor (eIF4G) of rice (*Oryza sativa*) associated with resistance to rice tungro spherical virus. Mol. Plant Microbe Interact..

[327] Wang D., Guo C.J., Huang J., Yang S.H., Tian D.C., Zhang X.H. (2014). Allele mining of rice blast resistance genes at AC134922 locus. Biochem. Biophy. Res. Commun..

[328] Hibino H., Ishikawa K., Omura T., Cabauatan P.Q., Koganezawa H. (1991). Characterization of rice tungro bacilliform and rice tungro spherical viruses. Phytopathology.

[329] Hibino H., Daquioag R.D., Mesina E.M., Aguiero V.M. (1990). Resistances in rice to tungro-associated viruses. Plant Dis..

[330] Chen J., Huang D.-R., Wang L., Liu G.J., Zhuang J.Y. (2010). Identificatiion of quantitative trait loci for resistance to whitebacked planthopper, Sogatella furcifera, from an interspecific cross *Oryza sativa  ×  O. rufipogon*. Breed. Sci..

[331] Hittalmani S., Parco A., Mew T.V., Zeigler R.S., Huang N. (2000). Fine mapping and DNA marker-assisted pyramiding of the three major genes for blast resistance in rice. Theor. Appl. Genet..

[332] Liu S.P., Li X., Wang C.Y., Li X.H., He Y.Q. (2002). Improvement of resistance to rice blast in Zhenshan 97 by molecular marker aided selection. Acta Bot. Sin..

[333] Narayanan N.N., Baisakh N., Cruz C.M.V., Gnanamanickam S.S., Datta K., Datta S.K. (2002). Molecular breeding for the development of blast and bacterial blight resistance in rice cv. IR50. Crop Sci..

[334] Lee S., Costanzo S., Jia Y., Olsen K.M., Caicedo A.L. (2009). Evolutionary dynamics of the genomic region around the blast resistance gene *Pi-ta* in AA genome Oryza species. Genetics.

[335] Singh A., Singh V.K., Singh S.P., Pandian R.T.P., Ellur R.K., Singh D., Bhowmick P.K., Gopala Krishnan S., Nagarajan M., Vinod K.K. (2012). Molecular breeding for the development of multiple disease resistance in Basmati rice. AoB Plants.

[336] Fu C.Y., Wu T., Liu W.G., Wang F., Li J.H., Zhu X.Y., Huang H.J., Liu Z.R., Liao Y.L., Zhu M.S. (2012). Genetic improvement of resistance to blast and bacterial blight of the elite maintainer line Rongfeng B in hybrid rice (*Oryza sativa* L.) by using marker-assisted selection. Afr. J. Biotechnol..

[337] Fukuoka S., Saka N., Mizukami Y., Koga H., Yamanouchi U., Yosuke Yoshioka Y., Hayashi N., Ebana K., Mizobuchi R., Yano M. (2015). Gene pyramiding enhances durable blast disease resistance in rice. Sci. Rep..

[338] Rafique M.Z., Zia M., Rashid H., Chaudhary M.F., Chaudhry Z. (2010). Comparison of transgenic plant production for bacterial blight resistance in Pakistani local rice (*Oryza sativa* L.) cultivars. Afr. J. Biotechnol..

[339] Qiu X., Pang Y., Yuan Z., Xing D., Xu J., Dingkuhn M., Li Z., Ye G. (2015). Genome-wide association study of grain appearance and milling quality in a worldwide collection of indica rice germplasm. PLoS ONE.

[340] McCouch S.R., Wright M.H., Tung C.W., Maron L.G., McNally K.L., Fitzgerald M., Singh N., DeClerck G., Agosto-Perez F., Korniliev P. (2016). Open access resources for genome-wide association mapping in rice. Nat. Commun..

[341] Raboin L.-M., Ballini E., Tharreau D., Ramanantsoanirina A., Frouin J., Courtois B., Ahmadi N. (2016). Association mapping of resistance to rice blast in upland field conditions. Rice.

[342] Abe A., Kosugi S., Yoshida K., Natsume S., Takagi H., Kanzaki H., Matsumura H., Yoshida K., Mitsuoka C., Tamiru M. (2012). Genome sequencing reveals agronomically-important loci in rice from mutant populations. Nat. Biotechnol..

[343] Yu H., Xie W., Li J., Zhou F., Zhang Q. (2014). A whole-genome SNP array (RICE6K) for genomic breeding in rice. Plant Biotechnol. J..

[344] Mgonja E.M., Park C.H., Kang H., Balimponya E.G., Opiyo S., Bellizzi M., Mutiga S.K., Rotich F., Ganeshan V.D., Mabagala R. (2017). Genotyping-by-sequencing-based genetic analysis of African rice cultivars and association mapping of blast resistance genes against *Magnaporthe oryzae* populations in Africa. Phytopathology.

[345] Zhang M.X., Xu J.L., Luo R.T., Shi D., Li Z.K. (2003). Genetic analysis and breeding use of blast resistance in a japonica rice mutant R917. Euphytica.

[346] Ahloowalia B.S., Maluszynski M., Nichterlein K. (2004). Global impact of mutation-derived varieties. Euphytica.

[347] Shu G.Y. (2009). Induced Plant Mutations in the Genomics Era.

[348] Alonso J.M., Ecker J.R. (2006). moving forward in reverse genetic technologies to enable genome-wide phenomic screens in *Arabidopsis*. Nat. Rev. Genet..

[349] Gurr S.J., Rushton P.J. (2005). Engineering plants with increased disease resistance: how are we going to express it?. Trends Biotechnol..

[350] Nordlee J.A., Taylor S.L., Townsend J.A., Thomas L.A., Bush R.K. (1996). Identification of a Brazil-nut allergen in transgenic soybeans. N. Engl. J. Med..

[351] Marvier M., McCreedy C., Regetz J., Kareiva P. (2007). A meta-analysis of effects of Bt cotton and maize on nontarget invertebrates. Science.

[352] Radosevich S.R., Ghersa C.M., Comstock G. (1992). Concerns a weed scientist might have about herbicide-tolerant crops. Weed Technol..

[353] Jepson P.C., Croft B.A., Pratt G.E. (1994). Test systems to determine the ecological risks posed by toxin release from *Bacillus thuringiensis* genes in crop plants. Mol. Ecol..

[354] Xiao N., Wu Y., Pan C., Yu L., Chen Y., Liu G., Li Y., Zhang X., Wang Z., Dai Z. (2017). Improving of rice blast resistances in japonica by pyramiding major R genes. Front. Plant Sci..

[355] Wang Z., Yano M., Yamanouchi U., Iwamoto M., Monna L., Hayasaka H., Katayose Y., Sasaki T. (1999). The *Pib* gene for rice blast resistance belongs to the nucleotide binding and leucine rich repeat class of plant disease resistance genes. Plant J..

[356] Lee S.-K., Song M.-Y., Seo Y.-S., Kim H.-K., Ko S., Cao P.-J., Suh J.-P., Yi G., Roh J.-H., Lee S. (2009). Rice *Pi5*-mediated resistance to *Magnaporthe oryzae* requires the presence of two coiled-coil–nucleotide-binding–leucine-rich repeat genes. Genetics.

[357] Fukuoka S., Saka N., Koga H., Ono K., Shimizu T., Ebana K., Hayashi N., Takahashi A., Hirochika H., Okuno K. (2009). Loss of function of a proline-containing protein confers durable disease resistance in rice. Science.

[358] Rai A.K., Kumar S.P., Gupta S.K., Gautam N., Singh N.K., Sharma T.R. (2011). Functional complementation of rice blast resistance gene *Pi-k^h^* (*Pi54*) conferring resistance to diverse strains of *Magnaporthe oryzae*. J. Plant Biochem. Biotechnol..

[359] Zhai C., Lin F., Dong Z., He X., Yuan B., Zeng X., Wang L., Pan Q. (2011). The isolation and characterization of *Pik*, a rice blast resistance gene which emerged after rice domestication. New Phytol..

[360] Ashikawa I., Hayashi N., Yamane H., Kanamori H., Wu J., Matsumoto T., Ono K., Yano M. (2008). Two adjacent nucleotide-binding site–leucine-rich repeat class genes are required to confer *Pikm*-specific rice blast resistance. Genetics.

[361] Yuan B., Zhai C., Wang W., Zeng X., Xu X., Hu H., Lin F., Wang L., Pan Q. (2011). The *Pik-p* resistance to *Magnaporthe oryzae* in rice is mediated by a pair of closely linked CC-NBS-LRR genes. Theor. Appl. Genet..

[362] Kumari M., Rai A.K., Devanna B.N., Singh P.K., Kapoor R., Rajashekara H., Prakash G., Sharma V., Sharma T.R. (2017). Co-transformation mediated stacking of blast resistance genes *Pi54* and *Pi54rh* in rice provides broad spectrum resistance against *Magnaporthe oryzae*. Plant Cell Rep..

[363] Quilis J., LopezGarcia B., Meynard D., Guiderdoni E., San Segundo B. (2014). Inducible expression of a fusion gene encoding two proteinase inhibitors leads to insect and pathogen resistance in transgenic rice. Plant Biotechnol. J..

[364] Sripriya R., Parameswari C., Veluthambi K. (2017). Enhancement of sheath blight tolerance in transgenic rice by combined expression of tobacco osmotin (*ap24*) and rice chitinase (*chi11*) genes. In Vitro Cell. Dev. Biol..

[365] Li H., Zhou S.-Y., Zhao W.-S., Su S.-C., Peng Y.-L. (2009). A novel wall-associated receptor-like protein kinase gene, *OsWAK1*, plays important roles in rice blast disease resistance. Plant Mol. Biol..

[366] Jha S., Tank H.G., Prasad B.D., Chattoo B.B. (2009). Expression of *Dm-AMP1* in rice confers resistance to *Magnaporthe oryzae* and *Rhizoctonia solani*. Transgenic Res..

[367] Helliwell E.E., Wang Q., Yang Y. (2013). Transgenic rice with inducible ethylene production exhibits broad spectrum disease resistance to the fungal pathogens *Magnaporthe oryzae* and *Rhizoctonia solani*. Plant Biotechnol. J..

[368] Molla K.A., Karmakar S., Chanda P.K., Sarkar S.N., Datta S.K., Datta K. (2016). Tissue-specific expression of *ArabidopsisNPR1* gene in rice for sheath blight resistance without compromising phenotypic cost. Plant Sci..

[369] Richa K., Tiwari I.M., Devanna B.N., Botella J.R., Sharma V., Sharma T.R. (2017). Novel chitinase gene LOC_Os11g47510 from indica rice Tetep provides enhanced resistance against sheath blight pathogen *Rhizoctonia solani* in rice. Front. Plant Sci..

[370] Datta K., Velazhahan R., Oliva N., Ona I., Mew T., Khush G.S., Muthukrishnan S., Datta S.K. (1999). Over-expression of the cloned rice thaumatin-like protein (PR-5) gene in transgenic rice plants enhances environmental friendly resistance to *Rhizoctonia solani* causing sheath blight disease. Theor. Appl. Genet..

[371] Mao B., Liu X., Hu D., Li D. (2014). Co-expression of *RCH10* and *AGLU1* confers rice resistance to fungal sheath blight *Rhizoctonia solani* and blast *Magnorpathe oryzae* and reveals impact on seed germination. World J. Microbiol. Biotechnol..

[372] Bundo M., Coca M. (2016). Enhancing blast disease resistance by overexpression of the calcium dependent protein kinase *OsCPK4* in rice. Plant Biotechnol. J..

[373] Bundo M., Coca M. (2017). Calcium-dependent protein kinase *OsCPK10* mediates both drought tolerance and blast disease resistance in rice plants. J. Exp. Bot..

[374] Li Y., Lu Y.-G., Shi Y., Wu L., Xu Y.-J., Huang F., Guo X.-Y., Zhang Y., Fan J., Zhao J.-Q. (2014). Multiple rice microRNAs are involved in immunity against the blast fungus *Magnaporthe oryzae*. Plant Physiol..

[375] Li Y., Zhao S.-L., Li J.-L., Hu X.-H., Wang H., Cao X.-L., Xu Y.-J., Zhao Z.-X., Xiao Z.-Y., Yang N. (2017). Osa-miR169 negatively regulates rice immunity against the blast fungus *Magnaporthe oryzae*. Front. Plant Sci..

[376] Campo S., Peris Peris C., Sire C., Moreno A.B., Donaire L., Zytnicki M., Notredame C., Llave C., San Segundo B. (2013). Identification of a novel microRNA (miRNA) from rice that targets an alternatively spliced transcript of the Nramp6 (Natural resistance associated macrophage protein 6) gene involved in pathogen resistance. New Phytol..

[377] Wang R., Lu L., Pan X., Hu Z., Ling F., Yan Y., Liu Y., Lin Y. (2015). Functional analysis of *OsPGIP1* in rice sheath blight resistance. Plant Mol. Biol..

[378] Maeda S., Hayashi N., Sasaya T., Mori M. (2016). Overexpression of *BSR1* confers broad-spectrum resistance against two bacterial diseases and two major fungal diseases in rice. Breed. Sci..

[379] Xue X., Cao Z.X., Zhang X.T., Wang Y., Zhang Y.F., Chen Z.X., Pan X.B., Zuo S.M. (2016). Overexpression of *OsOSM1* enhances resistance to rice sheath blight. Plant Dis..

[380] Chen X.J., Chen Y., Zhang L.N., Xu B., Zhang J.H., Chen Z.X., Tong Y.H., Zuo S.M., Xu J.Y. (2016). Overexpression of *OsPGIP1* enhances rice resistance to sheath blight. Plant Dis..

[381] Luan Z.H., Zhou D.W. (2015). Screening of rice (*Oryza sativa* L.) *OsPR1b*-interacting factors and their roles in resisting bacterial blight. Genet. Mol. Res..

[382] Zhao S., Hong W., Wu J., Wang Y., Ji S., Zhu S., Wei C., Zhang J., Li Y. (2017). A viral protein promotes host SAMS1 activity and ethylene production for the benefit of virus infection. eLife.

[383] Zhang D., Liu M., Tang M., Dong B., Wu D., Zhang Z., Zhou B. (2015). Repression of microRNA biogenesis by silencing of OsDCL1 activates the basal resistance to *Magnaporthe oryzae* in rice. Plant Sci..

[384] Wu J., Yang R., Yang Z., Yao S., Zhao S., Wang Y., Li P., Song X., Jin L., Zhou T. (2017). ROS accumulation and antiviral defence control by microRNA528 in rice. Nat. Plants.

[385] Wu J., Yang Z., Wang Y., Zheng L., Ye R., Ji Y., Zhao S., Ji S., Liu R., Xu L. (2015). Viral-inducible Argonaute18 confers broad-spectrum virus resistance in rice by sequestering a host microRNA. eLife.

[386] Sun Y., Zhang X., Wu C., He Y., Ma Y., Hou H., Guo X., Du W., Zhao Y., Xia L. (2016). Engineering herbicide-resistant rice plants through CRISPR/Cas9-mediated homologous recombination of acetolactate synthase. Mol. Plant.

[387] Zhou J., Peng Z., Long J., Sosso D., Liu B., Eom J., Huang S., Liu S., Vera Cruz C., Frommer W.B. (2015). Gene targeting by the TAL effector PthXo2 reveals cryptic resistance gene for bacterial blight of rice. Plant J..

[388] Wang Z., Han Q., Zi Q., Lv S., Qiu D., Zeng H. (2017). Enhanced disease resistance and drought tolerance in transgenic rice plants overexpressing protein elicitors from *Magnaporthe oryzae*. PLoS ONE.

[389] Hong Y., Yang Y., Zhang H., Huang L., Li D., Song F. (2017). Overexpression of *MoSM1*, encoding for an immunity-inducing protein from *Magnaporthe oryzae*, in rice confers broad-spectrum resistance against fungal and bacterial diseases. Sci. Rep..

[390] Tiwari I.M., Jesuraj A., Kamboj R., Devanna B.N., Botella J.R., Sharma T.R. (2017). Host delivered RNAi, an efficient approach to increase rice resistance to sheath blight pathogen (*Rhizoctonia solani*). Sci. Rep..

[391] Rajesh T., Maruthasalam S., Kalpana K., Poovannan K., Kumar K.K., Kokiladevi E., Sudhakar D., Samiyappan R., Balasubramanian P. (2016). Stability of sheath blight resistance in transgenic ASD16 rice lines expressing a rice chi11 gene encoding chitinase. Biol. Plant.

[392] Jiang W., Zhou H., Bi H., Fromm M., Yang B., Weeks D.P. (2013). Demonstration of CRISPR/Cas9/sgRNA-mediated targeted gene modification in *Arabidopsis*, tobacco, sorghum and rice. Nucleic Acids Res..

[393] Li T., Liu B., Spalding M.H., Weeks D.P., Yang B. (2012). High-efficiency TALEN-based gene editing produces disease-resistant rice. Nat. Biotechnol..

[394] Wang F., Wang C., Liu P., Lei C., Hao W., Gao Y., Liu Y.-G., Zhao K. (2016). Enhanced rice blast resistance by CRISPR/Cas9-targeted mutagenesis of the ERF transcription factor gene OsERF922. PLoS ONE.

[395] Sprague S.J., Balesdent M.-H., Brun H., Hayden H.L., Marcroft S.J., Pinochet X., Rouxel T., Howlett B.J. (2006). Major gene resistance in *Brassica napus* (oilseed rape) is overcome by changes in virulence of populations of *Leptosphaeria maculans* in France and Australia. Eur. J. Plant Pathol..

[396] Kumari M., Devanna B.N., Singh P.K., Rajashekara H., Sharma V., Sharma T.R. (2018). Stacking of blast resistance orthologue genes in susceptible indica rice line improves resistance against *Magnaporthe oryzae*. 3 Biotech.

[397] Lopez M.A., Bannenberg G., Castresana C. (2008). Controlling hormone signaling is a plant and pathogen challenge for growth and survival. Curr. Opin. Plant Biol..

[398] Grant M.R., Jones J.D.G. (2009). Hormone (dis) harmony moulds plant health and disease. Science.

[399] Delteil A., Zhang J., Lessard P., Morel J.-B. (2010). Potential candidate genes for improving rice disease resistance. Rice.

[400] Fire A., Xu S., Montgomery M.K., Kostas S.A., Driver S.E., Mello C.C. (1998). Potent and specific genetic interference by double-stranded RNA in *Caenorhabditis elegans*. Nature.

[401] Meister G., Tuschl T. (2004). Mechanisms of gene silencing by double-stranded RNA. Nature.

[402] Borges F., Martienssen R.A. (2015). The expanding world of small RNAs in plants. Nat. Rev. Mol. Cell Biol..

[403] Wagh S.G., Alam M.M., Kobayashi K., Yaeno T., Yamaoka N., Toriba T., Hirano H.-Y., Nishiguchi M. (2016). Analysis of rice RNA-dependent RNA polymerase 6 (*OsRDR6*) gene in response to viral, bacterial and fungal pathogens. J. Gen. Plant Pathol..

[404] Kamthan A., Chaudhuri A., Kamthan M., Datta A. (2015). Small RNAs in plants: Recent development and application for crop improvement. Front. Plant Sci..

[405] Nowara D., Gay A., Lacomme C., Shaw J., Ridout C., Douchkov D., Hensel G., Kumlehn J., Schweizer P. (2010). HIGS: Host-induced gene silencing in the obligate biotrophic fungal pathogen *Blumeria graminis*. Plant Cell.

[406] Zhu L., Zhu J., Liu Z., Wang Z., Zhou C., Wang H. (2017). Host-induced gene silencing of rice blast fungus *Magnaporthe oryzae* pathogenicity genes mediated by the brome mosaic virus. Genes.

[407] Zhang Y., Zhang F., Li X., Baller J.A., Qi Y., Starker C.G., Bogdanove A.J., Voytas D.F. (2013). Transcription activator-like effector nucleases enable efficient plant genome engineering. Plant Physiol..

[408] Cermak T., Baltes N.J., Cegan R., Zhang Y., Voytas D.F. (2015). High-frequency, precise modification of the tomato genome. Genome Biol..

[409] Wolt J.D., Wang K., Sashital D., Lawrence-Dill C.J. (2016). Achieving plant CRISPR targeting that limits off-target effects. Plant Genome.

[410] Zhao H., Wolt J.D. (2017). Risk associated with off-target plant genome editing and methods for its limitation. Emerg. Top. Life Sci..

[411] Zischewski J., Fischer R., Bortesi L. (2017). Detection of on-target and off-target mutations generated by CRISPR/Cas9 and other sequence-specific nucleases. Biotechnol. Adv..

[412] Yin Z., Chen J., Zeng L., Goh M., Leung H. (2000). Characterizing rice lesion mimic mutants and identifying a mutant with broad spectrum resistance to rice blast and bacterial blight. Mol. Plant Microbe Interact..

